# The Identification and Analysis of Novel Umami Peptides in Lager Beer and Their Multidimensional Effects on the Sensory Attributes of the Beer Body

**DOI:** 10.3390/foods14152743

**Published:** 2025-08-06

**Authors:** Yashuai Wu, Ruiyang Yin, Liyun Guo, Yumei Song, Xiuli He, Mingtao Huang, Yi Ren, Xian Zhong, Dongrui Zhao, Jinchen Li, Mengyao Liu, Jinyuan Sun, Mingquan Huang, Baoguo Sun

**Affiliations:** 1 School of Food Science and Engineering, South China University of Technology, Guangzhou 510640, China; wyss995418706@163.com (Y.W.); huangmt@scut.edu.cn (M.H.); 2 Beijing Key Laboratory of Beer Brewing Technology, Technology Center of Beijing Yanjing Beer Co., Ltd., Beijing 101300, China; ruiyang_yin@163.com (R.Y.); yanjing6089@163.com (L.G.); songym@163.com (Y.S.); niangjiu19@163.com (X.H.); 3 China Food Flavor and Nutrition Health Innovation Center, Beijing Technology and Business University, Beijing 100048, China; lijinchen@btbu.edu.cn (J.L.); lmy040024@163.com (M.L.); sunjinyuan@btbu.edu.cn (J.S.); huangmq@th.btbu.edu.cn (M.H.); sunbg@btbu.edu.cn (B.S.); 4 Key Laboratory of Brewing Molecular Engineering of China Light Industry, Beijing Technology & Business University, Beijing 100048, China; 5 Beijing Laboratory of Food Quality and Safety, Beijing Technology and Business University, Beijing 100048, China; 6 Beijing Changping District Food and Drug Safety Surveillance Center, Beijing Changping District Market Supervision Administration, Beijing 102200, China; 409695694ryan@sina.com; 7 School of Brewing Engineering, Moutai Institute, Renhuai 564501, China; zhongxian2025@163.com

**Keywords:** lager beer, umami peptide, single-factor sensory test, umami-oriented beer, off-flavor

## Abstract

This study was designed to systematically identify novel umami peptides in lager beer, clarify their molecular interactions with the T1R1/T1R3 receptor, and determine their specific effects on multidimensional sensory attributes. The peptides were characterized by LC-MS/MS combined with de novo sequencing, and 906 valid sequences were obtained. Machine-learning models (UMPred-FRL, Tastepeptides-Meta, and Umami-MRNN) predicted 76 potential umami peptides. These candidates were docked to T1R1/T1R3 with the CDOCKER protocol, producing 57 successful complexes. Six representative peptides—KSTEL, DELIK, DIGISSK, IEKYSGA, DEVR, and PVPL—were selected for 100 ns molecular-dynamics simulations and MM/GBSA binding-energy calculations. All six peptides stably occupied the narrow cleft at the T1R1/T1R3 interface. Their binding free energies ranked as DEVR (−44.09 ± 5.47 kcal mol^−1^) < KSTEL (−43.21 ± 3.45) < IEKYSGA (−39.60 ± 4.37) ≈ PVPL (−39.53 ± 2.52) < DELIK (−36.14 ± 3.11) < DIGISSK (−26.45 ± 4.52). Corresponding taste thresholds were 0.121, 0.217, 0.326, 0.406, 0.589, and 0.696 mmol L^−1^ (DEVR < KSTEL < IEKYSGA < DELIK < PVPL < DIGISSK). TDA-based sensory validation with single-factor additions showed that KSTEL, DELIK, DEVR, and PVPL increased umami scores by ≈21%, ≈22%, ≈17%, and ≈11%, respectively, while DIGISSK and IEKYSGA produced marginal changes (≤2%). The short-chain peptides thus bound with high affinity to T1R1/T1R3 and improved core taste and mouthfeel but tended to amplify certain off-flavors, and the long-chain peptides caused detrimental impacts. Future formulation optimization should balance flavor enhancement and off-flavor suppression, providing a theoretical basis for targeted brewing of umami-oriented lager beer.

## 1. Introduction

Lager beer is regarded as one of the most consumed and widely accepted alcoholic beverages worldwide. Its refreshing mouthfeel and balanced malt and hop aromas are favored by consumers. Moderate beer consumption can deliver several functional constituents—malt-derived B-vitamins, silicon, and soluble β-glucans, together with hop and malt polyphenols that exhibit antioxidant and anti-inflammatory activity—collectively linked to improved endothelial function, enhanced bone-mineral density, and a more diverse gut microbiota. These putative benefits, however, depend on responsible intake levels that avoid the well-documented risks of excessive alcohol consumption [1,2,3,4,5]. By 2024, the global beer market value was about USD 804.65 billion. Lager categories—including pale, Vienna, and dark styles—accounted for 86.46% of the total volume, corresponding to roughly USD 695.70 billion, and the amount was projected to rise to USD 898.149 billion by 2030, representing a compound annual growth rate (CAGR) of around 4.85% [1,2]. In preceding years, the beer consumption market was observed to have undergone clear structural differentiation. Contrasting development trends were shown in traditional industrial lager beer and emerging categories, such as craft and low-alcohol products. Although lager still occupied the dominant share of the market, accounting for about 90% of total sales, premium lager was reported to have achieved a rapid growth of 22% (https://www.hangyan.co/charts/3074591479960700641, https://economy.china.com/industrial/11173306/20180109/31933055_1.html, accessed on 29 July 2025), indicating an evident upgrade in consumption. At the same time, the craft beer market was noted to be flourishing. Its consumption in 2025 was projected to reach 2.3 billion liters, with a compound annual growth rate of 17% (https://www.tjkx.com/news/show/1097386, accessed on 29 July 2025). Craft lager was identified as one of the fastest-growing subcategories. Consumption scenarios and consumer groups were found to be significantly diversified. Industrial lager mainly relied on traditional festive social occasions, whereas craft products were better suited to home drinking and night-market settings. Generation Z contributed 65% of craft sales (https://m.163.com/dy/article/K4HFOMMT0522BL6H.html, accessed on 29 July 2025). The market exhibited a transformation towards reduced volume but enhanced quality. On one hand, a low-price strategy accelerated the popularization of craft beer; for example, the price of a 1 L craft pack at Hema was reduced to CNY 13.9 (http://www.itbear.com.cn/html/2025-07/896056.html, accessed on 29 July 2025). On the other hand, differentiated products, such as new Chinese-style craft lager and low-alcohol beverages, whose online sales grew by 28% (https://big5.chinabgao.com/freereport/105082.html, accessed on 29 July 2025), were widely welcomed, driving the industry toward higher quality and greater diversification. As purchasing power increased, a fundamental change in consumer demand for lager beer was observed, with preference shifting from “low price and ample quantity” to “moderate price and superior quality” [3,4].

Accordingly, the enhancement of lager beer quality was regarded as a shared objective within the industry. The abundant CO_2_ and mild alcohol content acted in concert to provide drinkers with a “constriction-ease” sense of physical and mental relaxation, while sour, sweet, and bitter tastes were fully expressed through interactions among malt, hops, and yeast secondary metabolites [5,6,7]. As investigations into taste dimensions advanced, it was observed that beer, like other foods, contained the fifth basic taste—umami—which was gradually considered an essential component of lager beer quality [8]. Nevertheless, mechanistic elucidation of umami characteristics in lager beer has remained at an early stage, and the molecular basis of this taste in lager beer has yet to be precisely clarified.

Umami in diverse food systems is usually formed by several small molecules, including free amino acids, such as L-glutamic acid, L-aspartic acid, and their sodium salts, 5′-nucleotides (IMP, GMP), organic acids, carboxylic acids, and low-molecular-weight peptides [9,10,11,12,13]. When raw materials undergo fermentation, enzymatic hydrolysis, or thermal processing, these precursors are converted into umami molecules, thereby laying the foundation for the savory taste of soups, sauces, and fermented alcoholic beverages [14]. During saccharification, yeast fermentation, and maturation, beer likewise accumulates these umami-active substances [15]. Such potential “umami factors” offer possibilities for exploring the distinctive refreshing taste of beer [16,17]. Growing evidence demonstrates that proteolysis during malting and fermentation generates a rich pool of short, glutamate- and aspartate-enriched peptides that can contribute directly to savory flavor in alcoholic beverages. An early LC-MS/MS survey catalogued more than 200 low-molecular-weight peptides in commercial barley–malt beers, several bearing acidic motifs compatible with T1R1/T1R3 activation [7]. Building on this, Schmidt et al. [17] showed that beers, wines, and champagnes aged on lees accumulate both free glutamate and small peptides, resulting in markedly higher “umami potential” than freshly fermented counterparts. Most recently, Huang et al. [16] combined high-resolution peptidomics with molecular docking and taste dilution analysis to identify a suite of lager-beer peptides—such as KSTEL and DELIK—that bind T1R1/T1R3 with sub-micromolar affinity and significantly elevate umami intensity. Together, these studies establish fermentation- and malting-driven peptide formation as mechanistic routes to umami enhancement in alcoholic beverages. Among these factors, umami peptides have attracted growing attention because of their low taste thresholds and pronounced umami expression [18,19,20]. These peptides, composed of a few amino acid residues linked by peptide bonds, generally possess molecular weights below 3 kDa and present advantages of natural origin, safety, and high nutritional value. They are key contributors to the “mellow and refreshing” attributes of many foods. Six octapeptides, including AEEHVEAVN, were isolated by Zhang et al. [14] from chicken-breast soup. Their umami thresholds ranged from 0.18 to 0.91 mmol L^−1^, equivalent to 0.53–0.66 g L^−1^ monosodium glutamate, and the peptides markedly enhanced the soup’s savory intensity. Yue et al. applied an enzymolysis–membrane separation strategy to identify 52 novel umami peptides and used molecular docking to show that these peptides stably bind sites, such as ASP-30 and MET-342, on the T1R1/T1R3 receptor, thereby revealing the mechanism underlying strong umami perception [21]. Comparative studies have also confirmed that higher peptide contents in enzymatic chicken broths produced more pronounced savory enhancement [22]. Since the discovery of the classical beef octapeptide, more than 300 umami peptides derived from fermented foods have been identified, and some originated from beer and other fermented alcoholic beverages [23]. Recent mass-spectrometry-based flavouromics research has located several novel umami peptides in lager beer and attempted to clarify their binding mechanisms with taste receptors through molecular docking and dynamic simulations, thereby providing theoretical support for targeted brewing [16].

The complex lager–beer matrix poses challenges for researchers’ extraction, isolation, and identification of umami peptides. Researchers’ identification of umami peptides has traditionally relied mainly on gel-filtration chromatography (GFC) and reversed-phase high-performance liquid chromatography coupled with mass spectrometry (HPLC-MS). These approaches possess clear limitations—long processing times, high costs, and low throughput—that have severely restricted progress in research on beer-derived umami peptides. To overcome these bottlenecks, computer-assisted peptide-identification techniques have been increasingly regarded as advantageous. Researchers have markedly improved umami-peptide identification efficiency, especially when they combine machine-learning techniques with in silico bioinformatics. Prediction tools such as UMPred-FRL, Umami-MRNN, and Tastepeptides-Meta have been applied to preliminary screening and threshold prediction of umami peptides and have demonstrated high efficiency and accuracy. For example, Qi et al. trained an MLP–RNN dual model on six categories of peptide-sequence features from 499 samples and achieved 90.5% accuracy in independent umami-property prediction tests [8]. In another study, a TPDM (taste peptide docking machine) was used as the core by Cui et al. [24], where residue-contact data from molecular docking, physicochemical descriptors, and Morgan fingerprints were integrated, and an ensemble weighted by an SVM over 19 high-performance sub-classifiers was constructed, enabling rapid and accurate discrimination between umami and bitter peptides. This “rapid screening before optimization” strategy highlighted the overall gain afforded by computer-assisted analysis over traditional methods and ensured the accuracy of subsequent research.

Based on, the polypeptides in lager beer were first screened by high-performance liquid chromatography coupled with quadrupole time-of-flight mass spectrometry. Potential umami peptides were then selected through umami–peptide prediction tools and molecular docking. The stability of peptide–receptor complexes was assessed by molecular dynamics simulations, and key umami peptides were predicted along with their molecular mechanisms and taste-expression characteristics. The threshold-determination and sensory-validation method was finally applied to verify these key peptides and to evaluate their multidimensional effects on beer sensory attributes.

## 2. Materials and Methods

### 2.1. Samples and Reagents

The experimental sample was a lager beer with an 8 °P wort concentration, produced from water, malt, rice, and hops, and was stored at −4 °C. Each treatment (control and six peptide-fortified beers) was brewed in three independent 500 L pilot batches (*n* = 3). The main reagents used in the experiment were acetonitrile (ACN) (Beijing InnoChem Science & Technology Co., Ltd., Beijing, China), formic acid (FA) (≥99%, chromatographic grade, Sigma-Aldrich, St. Louis, MO, USA), and ultrapure water. Six umami peptides—KSTEL, DELIK, DIGISSK, IEKYSGA, DEVR, and PVPL—were employed, each with a purity of ≥90% (Nanjing Taopu Biotechnology Co., Ltd., Nanjing, China).

### 2.2. Experimental Instruments

Major instruments and equipment: Milli-Q ultrapure water system (Milli-Q, Millipore, Billerica, MA, USA); 1000 µL pipette and 10 mL/100 mL volumetric flasks (Sinopharm Chemical Reagent Co., Beijing, China); 2 mL autosampler vials (Santa Clara, CA, USA); Retain-AX SPE cartridges (Waltham, MA, USA); GGC-C separatory–funnel vertical oscillator (Beijing Guohuan Hi-Tech Automation Technology Research Institute, Beijing, China); VM-500S vortex mixer (Joan Lab, Huzhou, Zhejiang, China); RE-52C rotary evaporator (Shanghai Yarong Biochemical Instrument Factory, Shanghai, China); SHB-III circulating-water vacuum pump (Zhengzhou Greatwall Scientific Industrial and Trade Co., Zhengzhou, China); Fresco 17 freeze dryer; UltiMate 3000 HPLC system; and Q Exactive high-resolution mass spectrometer (Thermo Scientific, Waltham, MA, USA).

### 2.3. Experimental Methods

#### 2.3.1. Preprocessing Method

A 10 mL beer sample was collected before being mixed with 10 mL loading buffer, a 2% acetonitrile aqueous solution containing 0.1% formic acid. Peptides were extracted and enriched by passage through a WCX SPE cartridge. The eluate was centrifugally concentrated and dried in preparation for LC-MS/MS analysis (n = 3).

#### 2.3.2. LC-MS/MS Analytical Conditions

Chromatographic conditions: The polypeptides were separated on a Waters Acquity Peptide C_18_ column (2.1 mm × 150 mm, 1.7 µm). Mobile phase A was 0.1% formic acid in water, and mobile phase B was 0.1% formic acid in acetonitrile. An injection volume of 20 µL was employed. The column temperature was maintained at 45 °C. Detection was performed at 215 nm and 280 nm. Gradient elution was applied according to the program listed in Table 1.

Mass spectrometry conditions: The samples were analyzed using a Q-Exactive high-resolution mass spectrometer in positive ion detection mode. The mass spectrometer ion source parameters are listed in Table 2. Mass spectrometry data were acquired in data-dependent acquisition (DDA) mode. The MS^1^ full-scan resolution was set to 70,000 (at *m*/*z* 200), with a scan range of 300–1500 *m*/*z*, a maximum injection time of 100 ms, and an AGC target value of 3 × 10^6^. In each MS^1^ acquisition cycle, the 10 strongest precursor ions (charge state 1+~5+) were selected, isolated within a 1.6 *m*/*z* window, and fragmented using high-energy collision-induced dissociation (HCD), with a collision energy setting of NCE = 28 eV. The resulting MS^2^ spectra were recorded at a resolution of 17,500 (*m*/*z* 200), with an AGC target value of 2 × 10^5^, a maximum injection time of 50 ms, and a dynamic exclusion time of 4 s to prevent repeated fragmentation of the same precursor ion.

#### 2.3.3. Qualitative Analysis of Peptides in Beer

The raw MS files were processed, and proteins were identified with PEAKS Studio v8.5 (Bioinformatics Solutions Inc., Waterloo, ON, Canada). Sequence searches were carried out against the protein databases for *Triticum aestivum*, *Komagataella phaffii*, and *Oryza sativa,* downloaded from UniProt. The parameters were set as follows: MS^1^ mass tolerance, 10 ppm; MS^2^ mass tolerance, 0.03 Da; digestion mode, none (unspecific); fixed modifications, none; and variable modifications, including protein N-terminal acetylation, deamidation (N/Q), oxidation (M), pyro-glutamate formation from glutamic acid (E) or glutamine (Q), and half disulfide (−1.01 Da). A confidence threshold of −10logP ≥ 15 was applied. To capture peptide segments possibly missing from the databases, the de novo sequencing function in PEAKS was employed to interpret the fragment spectra. The de novo results were evaluated by average local confidence (ALC), and only sequences with ALC ≥ 90% were retained to ensure reliability. These sequences were then used to complement and cross-validate the database search results during subsequent alignment and functional analyses [25,26,27,28,29,30,31,32].

#### 2.3.4. Efficient Screening Method for Potential Umami Peptides Using Machine Learning

UMPred-FRL (http://pmlabstack.pythonanywhere.com/UMPred-FRL, accessed on 29 July 2025) and Tastepeptides-Meta (http://tastepeptides-meta.com/TPDM, accessed on 29 July 2025) were preferentially employed to assess whether the polypeptides possessed umami activity. Probability values for umami activity were output. Thresholds predicted by Umami-MRNN (https://umami-mrnn.herokuapp.com/, accessed on 29 July 2025) were integrated with sensory evaluations to determine experimental thresholds and to support single-factor addition experiments.

#### 2.3.5. Molecular Docking Method

The sequence of the template protein mGluR1 was retrieved from UniProt-KB as a reference for homology modelling. The metabolotropic glutamate receptor (PDB ID: 1EWK, obtained from RCSB PDB, http://www.rcsb.org/, accessed on 29 July 2025) was adopted as the template. The amino acid sequences of umami-receptor subunits T1R1 and T1R3 were combined, and three-dimensional homology models were generated on the SwissModel platform (https://swissmodel.expasy.org/, accessed on 29 July 2025). After modelling, geometric reasonableness was examined with the Ramachandran plot, calculated by SAVES v6.0 (https://saves.mbi.ucla.edu/, accessed on 29 July 2025). The plot displayed φ–ψ dihedral-angle distributions and evaluated structural reliability. After validation, the model was submitted to molecular-docking studies. Before docking, the receptor structure was pre-processed in PyMOL 2.6.0 by removing all solvent molecules, ions, and small ligands. A docking grid was then set to cover the whole protein surface. Peptide ligands were constructed in Discovery Studio 2019 and were assigned CHARMm force-field parameters. Energy minimization was carried out with the Smart Minimizer algorithm (maximum 2000 steps; RMS-gradient threshold 0.01). Potential binding pockets were searched with the same software. After the binding sites had been defined, candidate umami peptides were embedded into the T1R1/T1R3 complex with the CDOCKER semi-flexible protocol. The other parameters were kept at default values, and only the pose with the highest CDOCKER-Energy score was retained. The resulting complex was visualized and analyzed in three dimensions with PyMOL and Discovery Studio to present ligand–receptor interactions intuitively [33,34,35,36,37,38].

#### 2.3.6. Determination Method of Sensory Threshold for Umami Peptides

The TDA taste-dilution analysis method [39,40,41] was applied to determine the peptides’ taste threshold. A stock solution of the target umami peptide was prepared at pH 6.5 and 1 mg mL^−1^. The stock was serially diluted with deionized water at a 1:1 ratio to create gradient samples. These samples were presented to a panel of twenty trained assessors in ascending concentration order. Each dilution was examined by the three-cup test, which contained two blanks and one sample. The assessors identified the differing cup and its lowest detectable concentration, and the result was confirmed through a repeat evaluation with the same set of samples.

#### 2.3.7. Molecular Dynamics (MDs) and MM/GBSA Binding Free Energy Calculation

The peptide–receptor complex obtained from docking was used as the initial conformation, and an all-atom molecular-dynamics simulation was carried out in AMBER 22 [42,43]. Both peptide chains and protein residues were parameterized with the ff14SB force field [44,45]. Hydrogen atoms were added with the LEaP tool, and a truncated-octahedral TIP3P water box was generated 10 Å from the system boundary [46,47,48], and Na^+^/Cl^−^ ions were introduced to maintain electrical neutrality. Topology and coordinate files were then exported for subsequent calculations. Energy minimization consisted of 2500 steps of steepest descent, followed by 2500 steps of conjugate gradient. The system was heated for 200 ps under constant volume, and the temperature was raised linearly from 0 K to 298.15 K. After temperature stabilization, a 500 ps NVT equilibration was performed to promote uniform solvent distribution. The ensemble was next switched to NPT and pre-equilibrated for another 500 ps. A 100 ns NPT production run was finally executed under periodic boundary conditions. Simulation settings were as follows. The non-bonded interaction cut-off was 10 Å. Long-range electrostatics were treated with the particle-mesh Ewald method [49,50,51]. All bonds involving hydrogen were constrained by SHAKE [52]. The temperature was controlled with Langevin dynamics at a collision frequency of γ = 2 ps^−1^ [53]. The pressure was kept at 1 atm. The integration time step was 2 fs. Trajectories were saved every 10 ps for later structural and energetic analyses.

Binding free energies between proteins and ligands in all systems were evaluated with the MM/GBSA method [54,55,56,57,58]. Because extended trajectories might reduce MM/GBSA accuracy [55,56], frames from 90–100 ns were adopted for the calculations, as expressed by the following equation:

(1) $$\Delta G_{bind}={\Delta G}_{\mathrm{complex}}-({\Delta G}_{\mathrm{receptor}}+\cdot {\Delta G}_{\mathrm{ligand}})={\Delta E}_{\mathrm{internal}}+{\Delta E}_{\mathrm{vDW}}+{\Delta E}_{\mathrm{elec}}+{\Delta G}_{\mathrm{GB}}+{\Delta G}_{\mathrm{SA}}$$

In Equation (1), ΔE_internal_ was defined as the internal energy, ΔEVDW as the van der Waals contribution, and ΔE_elec_ as the electrostatic interaction. The internal energy was composed of bond energy (E_bond_), angle energy (E_angle_), and torsional energy (E_torsion_). ΔGGB and ΔGSA were collectively termed the solvation free energy, where GGB represented the polar contribution and GSA the non-polar contribution. ΔGGB was calculated with the generalized Born model developed by Nguyen et al. [59] (igb = 2). The non-polar solvation free energy (ΔGSA) was obtained by multiplying the surface tension coefficient (γ) by the solvent-accessible surface area (SA), according to $\Delta G_{SA}=\cdot 0.0072\cdot \times \cdot \Delta \mathrm{SASA}$ (2). The entropy term was neglected because of its high computational cost and limited accuracy [54,57,58].

#### 2.3.8. Sample Sensory Evaluation and Single Addition Variable Method

Twenty milliliters of lager beer were accurately measured for each sample. The original sample was labelled A. Samples prepared by individually adding the umami peptides KSTEL, DELIK, DIGISSK, IEKYSGA, DEVR, and PVPL were labelled A-1, A-2, A-3, A-4, A-5, and A-6, respectively. Each peptide was added at 500 μL of its taste-threshold solution.

Sample A was first subjected to sensory evaluation. The descriptors and 0–9 quantitative standards for thirteen key sensory attributes of lager beer are listed in Table 3, providing criteria for assessing aroma, flavor, and mouthfeel. The same sensory assessment was then applied to samples A-1 through A-6, and changes in the beer body after single additions of each umami peptide were compared.

A trained panel comprising 20 beer assessors (each with ≥120 h practice using flavor-reference standards) performed the sensory evaluation. Panel consistency was first verified on control batches via an ISO 4120 [60] triangle test; only assessors achieving ≥80% discrimination accuracy proceeded to the main study. Samples (30 mL) were served at 8 ± 1 °C in tulip glasses coded with random three-digit numbers and presented monadically under red light with unsalted crackers and water for palate cleansing. Quantitative descriptive analysis was then conducted, with each sensory attribute rated on a 9 cm unstructured line scale anchored at “not perceptible” (0) and “extremely intense” (9). Hedonic preference was assessed in a separate session using a 9-point hedonic scale (1 = dislike extremely, 9 = like extremely); it affords sufficient discriminatory power for acceptability judgements without imposing undue cognitive load on trained assessors.

Attribute selection adhered to the ISO 11035:1994 [61] two-stage procedure: the candidate descriptors were first compiled from established sources—including the ASBC Beer Flavor Wheel, the BJCP sensory lexicon, and recent lager-description studies—then evaluated in a focus-group session, where the twenty trained assessors tasted control and reference beers, discussed definitions, and anchored intensities with GRAS standard solutions [5,7,16]. Terms cited by at least 30% of panelists and exhibiting non-redundant semantic content were retained, yielding the 15 attributes presented in Table 3.

#### 2.3.9. Statistical Analysis

Origin 2021 software was used to draw radar diagrams and fingerprints; Tbtools was used to draw heatmaps; SPSS 24.0 software was applied for single-factor and correlation analysis; and the R software was used to extract and visualize the results of cluster analysis.

## 3. Results and Discussion

### 3.1. Qualitative Identification of Peptides in Lager Beer and Predictive Analysis of Potential Umami Peptides

Applying a confidence threshold of −10logP ≥ 15 for database-matched peptides and retaining only de novo sequences with ALC ≥ 90% secured adequate reliability, which yielded 906 peptides. Their umami activity was predicted with UMPred FRL and Tastepeptides-Meta. The qualitative parameters and predicted umami values for the lager-beer peptides are listed in the attached Table A1. In the machine-learning-identified pool of potential umami peptides, the peptide set showed a strong bias toward short chains: pentapeptides accounted for 48% of all sequences, tetrapeptides for 19%, and together with hexapeptides, these lengths composed about 81% of the total, whereas peptides longer than eight residues were scarce. Leucine-led starters were common—Leu, Thr, and Ala initiated over half of the sequences—and hydrophobic or small residues predominated overall. Leu (14.4%), Val (11.7%), Ala (11.2%), and Pro (9.6%) emerged as the four most frequent amino acids. Acidic side chains were present but less abundant, with Glu and Asp together contributing roughly 9% of all residues, while basic Lys and Arg each remained near 3%. These patterns suggested that umami-active peptides in lager beer had favored compact backbones rich in aliphatic residues.

### 3.2. Preliminary Screening of Umami Peptides and Molecular Docking Analysis

**Figure 1:**
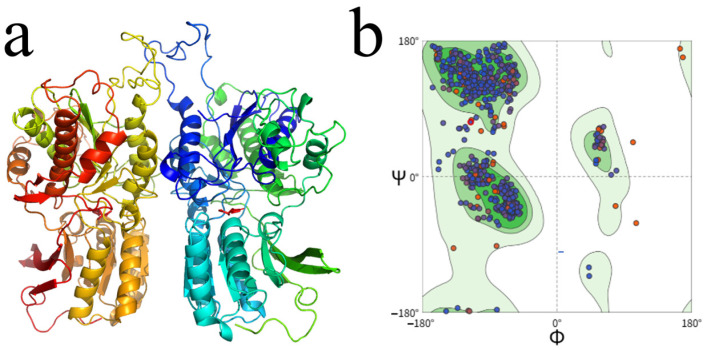
Homology modeling results for the taste receptor. (**a**) The homology modeling structure of the T1R1/T1R3 taste receptor. (**b**) A Ramachandran plot.

Potential umami peptides with both UMPred-FRL-Probability and ProUmami scores exceeding 0.7 were selected. A total of seventy-six small peptides were docked to the T1R1/T1R3 umami receptor for further screening. T1R1 and T1R3 formed a heterodimeric receptor whose extracellular Venus fly-trap (VFT) domains captured and fixed umami ligands. In the present work, the dimer was split into two separate subunits. Homology models were then built for each subunit, and their stereochemistry was examined with Ramachandran plots. Figure 1a shows a closed conformation for T1R1, whereas T1R3 remained open, creating a wide cavity capable of accommodating long-chain umami peptides. Although previous studies indicated that peptides mainly bound to T1R3, site-directed mutagenesis and simulations also demonstrated that T1R1 recognized small ligands, such as dipeptides, tripeptides, and amino acids. Both chains were therefore considered indispensable during taste recognition. Reliable structures can usually be obtained when sequence identity between target and template proteins reaches ≥30%. The identities of T1R1 and T1R3 with their templates were 34.34% and 33.55%, respectively, meeting this criterion. Ramachandran statistics (Figure 1b) indicated that 97.7% of residues lay in allowed regions, with 87.7% in the most favored regions, 10.0% in additionally allowed regions, and only 1.8% in generously allowed regions; residues in disallowed regions accounted for less than 0.5%. More than ninety per cent of φ–ψ angles, therefore, fell within a reasonable range, confirming that the models possessed good geometric quality and could serve as a reliable basis for subsequent docking and mechanistic studies.

Molecular docking was carried out with the semi-flexible CDOCKER algorithm in Discovery Studio. Other parameters were left at default values. Only the conformation with the lowest docking energy was retained. Fifty-seven peptides were finally docked successfully (Table 4). The group contained six tetrapeptides, thirty-six pentapeptides, eleven hexapeptides, and four heptapeptides. Thirty-one peptides contained aspartic acid (D) or glutamic acid (E). The D/E consensus effect served as an important criterion for selecting umami peptides.

Tighter peptide–receptor binding was indicated by lower docking energies. It was shown [9,10,11,13,62,63,64,65,66,67,68,69,70] that the N-terminus of the umami peptides was usually enriched in acidic amino acids (Asp, Glu) or small hydrophilic residues (Gly, Ala, Ser). Through carboxyl or other polar groups, those residues formed hydrogen bonds or electrostatic interactions with key receptor sites, such as Arg151 and His71 in T1R1, thereby activating the signaling pathway. The C-terminus tended to contain hydrophobic amino acids (Leu, Pro, Val) or polar residues (His, Gln). Hydrophobic side chains were inserted into the receptor’s hydrophobic pocket, exemplified by Tyr198 in T1R3, whereas polar residues further stabilized the interface. Umami intensity was markedly enhanced by the cooperative distribution of acidic (D/E) and basic (Arg, Lys) residues, because efficient receptor binding was achieved through charge complementarity between E/D and R/H. The umami peptides generally adopted β-turn-dominated secondary structures, a conformation that favored exposure of active sites.

On the basis of the preceding findings and the binding energies in Table 5, the peptides KSTEL, DELIK, DIGISSK, IEKYSGA, and DEVR were selected as potential umami candidates. PVPL, an atypical D/E-independent peptide enriched in hydrophobic residues (L/P/V), was also chosen. These six peptides were synthesized, their taste thresholds were measured (Table 5), molecular-dynamics simulations were conducted, and single-factor addition experiments were performed. Species–database matching indicated that these six peptides originated from *Triticum turgidum*, *Saccharomyces cerevisiae*, and *barley*, which aligned with lager-beer ingredients. Notably, except for PVPL, the other five peptides were newly identified and showed no matches in the sensory peptides and amino acids database (https://www.uwm.edu.pl/biochemia/index.php/pl/biopep, accessed on 29 July 2025). Their interaction mechanisms with taste receptors were investigated further in subsequent work.

### 3.3. Analysis of Binding Modes of 6 Types of Umami Peptides with Receptor Proteins

**Figure 2:**
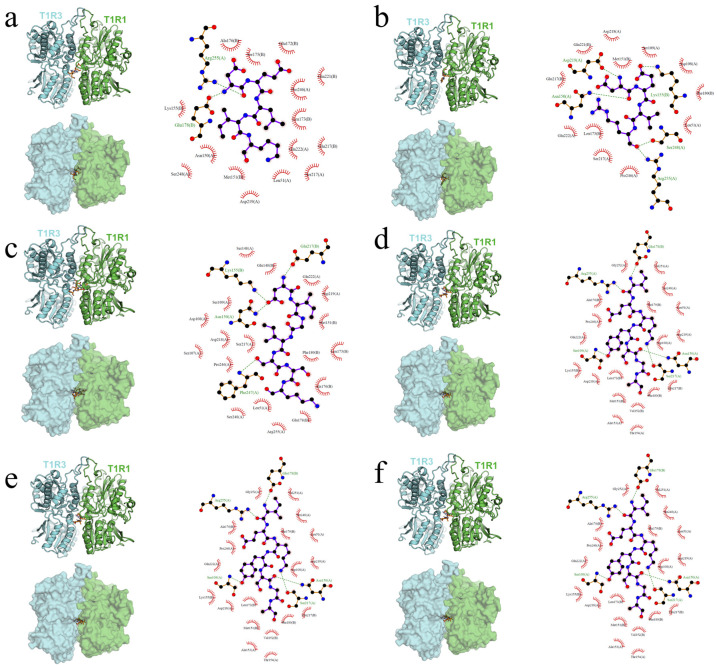
Molecular-docking diagrams. The left panel displays an overall view. The ligand is rendered as orange sticks. The T1R1 protein is shown in green, and the T1R3 protein is shown in cyan. The right panel displays a 2D interaction diagram. The dashed lines indicate hydrogen bonds. Chain A represents T1R1, and chain B represents T1R3. (**a**) The binding mode of T1R1–T1R3/DELIK, obtained by docking. (**b**) The binding mode of T1R1–T1R3/DEVR, obtained by docking. (**c**) The binding mode of T1R1–T1R3/DIGISSK, obtained by docking. (**d**) The binding mode of T1R1–T1R3/IEKYSGA, obtained by docking. (**e**) The binding mode of T1R1–T1R3/KSTEL, obtained by docking. (**f**) The binding mode of T1R1–T1R3/PVPL, obtained by docking.

As shown in Figure 2a, the DELIK molecule was embedded in the narrow cleft between the T1R1 (green) and T1R3 (cyan) subunits and bridged their interface. In the 2D interaction map, three main hydrogen bonds were observed. One connected the side-chain nitrogen of Arg255(A) in T1R1 to the ligand backbone. A second linked the carboxyl group of DELIK to Glu178(B) in T1R3. The hydrophobic side chains of the ligand contacted Leu51(A) in T1R1 and Met151(B), Ala176(B), and Ser175(B) in T1R3 through van der Waals forces. The combination of polar and hydrophobic contacts conferred high affinity and stability on the complex.

Figure 2b showed DEVR in the same cleft. Four key hydrogen bonds were detected. Bonds formed with Asp219(A), Asp150(A), and Ser248(A) in T1R1. An additional nitrogen–oxygen bond was involved in Arg255(A). The ligand amine also bonded to Lys155(B) in T1R3. Hydrophobic alignment with Leu173(A) and Pro246(A) in T1R1 and Ile180(B) and Gln217(B) in T1R3 reinforced binding.

Figure 2c displays DIGISSK as an orange rod lodged firmly in the cleft. Four hydrogen bonds were present. The N-terminal carboxyl bonded to Asn150(A) in T1R1. Further bonds linked Lys155(B) and Gln217(B) in T1R3 and the backbone nitrogen of Phe247(A) in T1R1. Surrounding hydrophobic residues—Leu51(A) in T1R1 and Ile151(B), Phe180(B), Ile173(B), and Ala176(B) in T1R3—created tight van der Waals packing.

As shown in Figure 2d, IEKYSGA occupied the cleft. Four hydrogen bonds anchored the ligand. Its carboxyl group bonded to Arg255(A) and Ser109(A) in T1R1. A mid-chain carbonyl bonded to Glu178(B) in T1R3. Additional bonds involved Asn150(A) and Ser217(A) in T1R1. Hydrophobic support came from Pro246(A), Thr154(A), and Ala153(A) in T1R1 and Ala176(B), Leu173(B), Met151(B), and Val152(B) in T1R3.

Figure 2e shows KSTEL in the same pocket. Three hydrogen bonds secured the ligand. The carboxyl group bonded to Glu217(B) in T1R3. Additional bonds linked Ser248(A) and Arg255(A) in T1R1. Hydrophobic residues—Leu51, Pro246, Phe247, and Val251 in T1R1, plus Phe180, Ile173, and Ala176 in T1R3—enveloped the peptide.

Figure 2f presents PVPL with two hydrogen bonds. One bonded to Asn150(A) in T1R1, and another to Gln221(B) in T1R3. The hydrophobic side chains were surrounded by Ile51(A) and Arg255(A) in T1R1 and Ile180(B), Leu173(B), and Met151(B) in T1R3.

The docking results for the six peptides indicated that the narrow, open-ended pocket between T1R1 and T1R3 acted as the common binding core. Each peptide bridged the two subunits. One end usually bonded to Arg255(A) in T1R1, while the other anchored to Glu178(B), Lys155(B), or Gln221(B) in T1R3, forming a cross-subunit polar clamp. A sheath of hydrophobic residues—Leu51, Ile/Leu173, Ala176, Met151, and Phe180—provided non-polar support. Longer peptides with richer polar side chains, such as DELIK, DEVR, DIGISSK, and IEKYSGA, formed more hydrogen bonds and showed stronger affinity and stability. Shorter peptides like PVPL relied on tight hydrophobic packing for notable binding. This polar-clamp plus hydrophobic-sheath mechanism, with Arg255(A), Lys155(B), and Glu178(B) as hotspot anchors, appeared to underlie umami–peptide recognition by the T1R1/T1R3 receptor. Molecular-dynamics simulations were subsequently carried out to validate binding strength and stability.

### 3.4. Molecular Dynamics Simulation Analysis

#### 3.4.1. Stability Analysis

**Figure 3:**
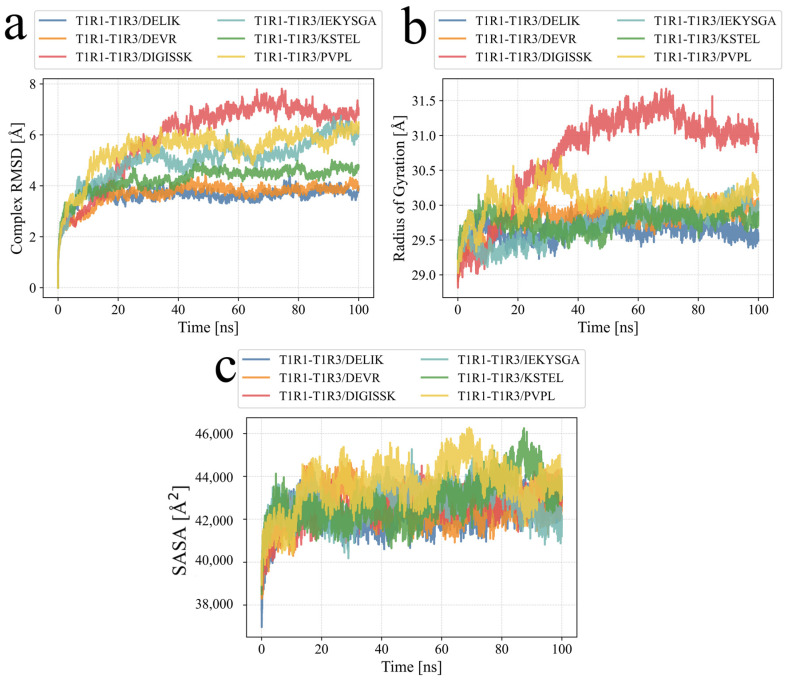
Stability analysis from molecular-dynamics simulations. (**a**) The root-mean-square deviation (RMSD) was tracked over time. (**b**) The radius of gyration (RoG) was monitored during the simulation. (**c**) The solvent-accessible surface area (SASA) was calculated for each complex.

As shown in Figure 3a, the RMSD values of all six complexes rose quickly during the first 10 ns and then reached plateaus. Each complex stabilized at a different level. T1R1–T1R3/DELIK settled at 3.5–4.0 Å and fluctuated least, indicating high conformational stability. T1R1–T1R3/DEVR remained at 3.8–4.5 Å. T1R1–T1R3/KSTEL stabilized near 4.5–5.0 Å, with moderate variation. T1R1–T1R3/IEKYSGA and T1R1–T1R3/PVPL drifted slowly between 5.0 and 6.5 Å, implying some interfacial flexibility. T1R1–T1R3/DIGISSK showed the highest RMSD, 6.5–7.5 Å, and the largest fluctuations, reflecting greater conformational freedom and lower binding rigidity. Overall, DELIK and DEVR were bound more stably than the other peptides, whereas DIGISSK displayed the largest structural changes.

Figure 3b indicates that the radii of gyration (Rg) rose from an initial ~28.8 Å to equilibrium ranges within the first 10 ns and then became stable. After 10 ns, T1R1–T1R3/DELIK maintained the lowest Rg at ~29.3 Å, revealing the most compact complex. Equilibrium Rg values of ~29.5–29.9 Å with small fluctuations were recorded for T1R1–T1R3/DEVR, IEKYSGA, and KSTEL. PVPL fluctuated between ~29.6 and 30.3 Å, indicating moderate looseness. DIGISSK reached the highest Rg, stabilizing at ~30.8–31.2 Å after 40 ns, which signaled a more extended and flexible conformation. With the exception of DIGISSK, the complexes showed low Rg values at equilibrium and thus retained high structural compactness.

Figure 3c shows that the solvent-accessible surface areas (SASAs) climbed from ~37,000 Å^2^ to equilibrium ranges within about 10 ns, and then stabilized with ±1000 Å^2^ fluctuations. The lowest SASA of 40,500–41,500 Å^2^ was observed for T1R1–T1R3/DELIK, confirming its compact nature in water. IEKYSGA followed at 41,500–42,200 Å^2^. Intermediate values of ~42,000–43,000 Å^2^ and ~42,500–43,500 Å^2^ were recorded for DEVR and DIGISSK. KSTEL and PVPL exhibited the highest exposures, with PVPL reaching ~43,500–45,000 Å^2^, suggesting more open structures. The SASA ranking agreed with the Rg results: DELIK was most compact, whereas PVPL was most loosely packed.

**Figure 4:**
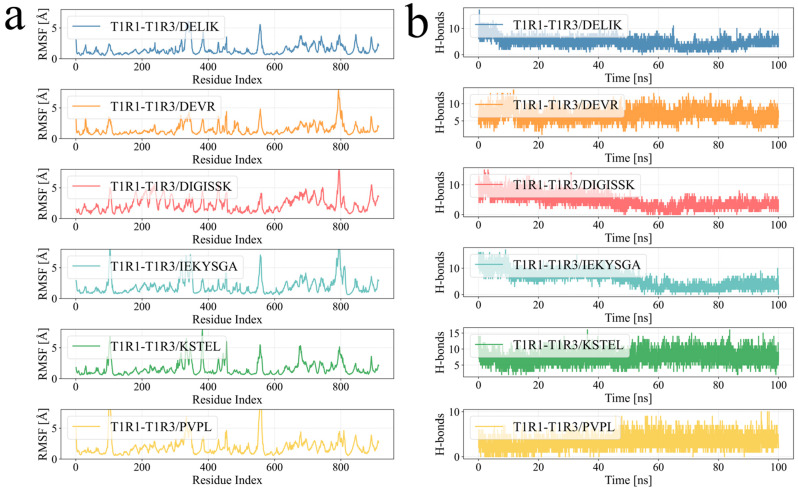
Molecular-dynamics simulation. (**a**) The root-mean-square fluctuation (RMSF) values were calculated from the trajectories. (**b**) The number of hydrogen bonds between each ligand and the protein was tracked during the simulation.

As illustrated in Figure 4a, the RMSF values were used to reflect protein flexibility during molecular-dynamics simulations. Drug binding usually reduces protein flexibility and thereby stabilizes the protein to support catalytic activity. After binding with the various small molecules, low RMSF values were observed across all proteins except at the two termini, indicating a rigid core.

According to Figure 4b, the number of hydrogen bonds in each complex underwent a rearrangement period during the first 5–10 ns and then reached a relatively stable phase, although clear differences appeared in average counts and stability. The T1R1–T1R3/KSTEL complex maintained the highest level, with about 8–10 hydrogen bonds and minimal fluctuations. T1R1–T1R3/DEVR followed at about 7–9 bonds. Counts for T1R1–T1R3/DELIK and IEKYSGA fell from 10–12 to 5–8 and 4–7, respectively, before showing slight recovery. T1R1–T1R3/DIGISSK dropped rapidly to 3–5 bonds, and later rose modestly to 4–6. T1R1–T1R3/PVPL showed the fewest hydrogen bonds, remaining near zero for the initial 10 ns and then increasing slowly to about 3–5. Overall, the KSTEL and DEVR systems formed the most numerous and stable polar interactions, whereas PVPL exhibited the sparsest hydrogen-bond network, indicating weaker polar contacts at the binding interface.

#### 3.4.2. MM-GBSA Binding Energy Results

**Figure 5:**
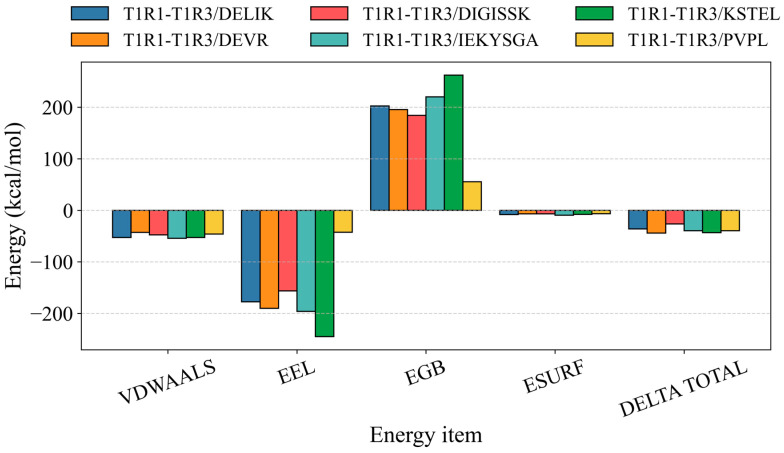
The MM-GBSA binding energies and energy decomposition were displayed.

Binding energies were calculated by the MM-GBSA method from molecular-dynamics trajectories, and the values more accurately reflected ligand–protein binding patterns. As shown in Table 6 and Figure 5, the binding energies of the T1R1–T1R3/DELIK, T1R1–T1R3/DEVR, T1R1–T1R3/DIGISSK, T1R1–T1R3/IEKYSGA, T1R1–T1R3/KSTEL, and T1R1–T1R3/PVPL complexes were −36.14 ± 3.11, −44.09 ± 5.47, −26.45 ± 4.52, −39.60 ± 4.37, −43.21 ± 3.45, and −39.53 ± 2.52 kcal mol^−1^, respectively. Negative values indicated binding affinity, and lower values signified stronger interactions. The calculations, therefore, demonstrated appreciable affinity between each ligand and the receptor.

### 3.5. The Validation Experiment Performed with the Single-Factor Addition Method

#### 3.5.1. Sensory-Enhancement Effects of Umami Peptides and Analysis of Their Structure–Function Relationships

**Figure 6:**
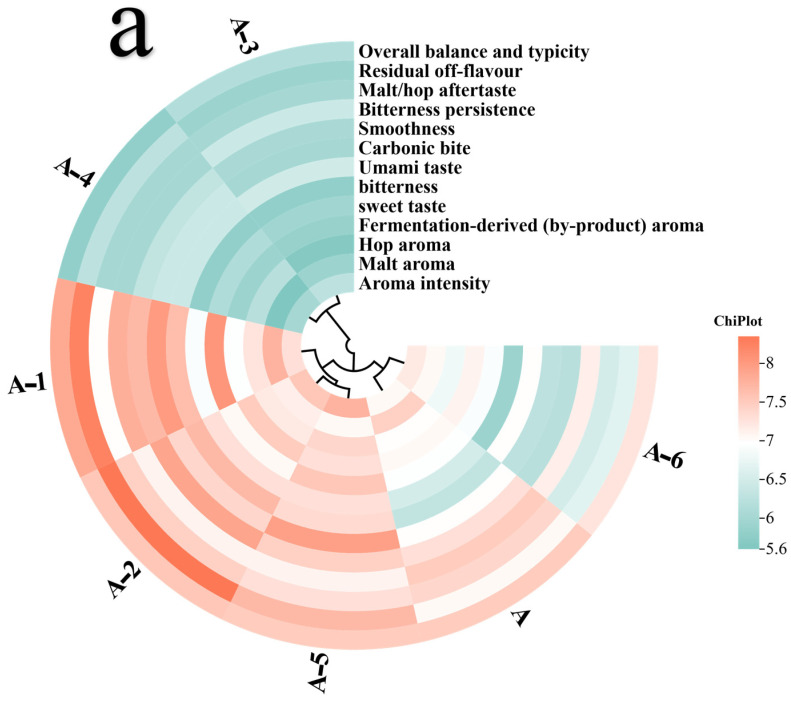

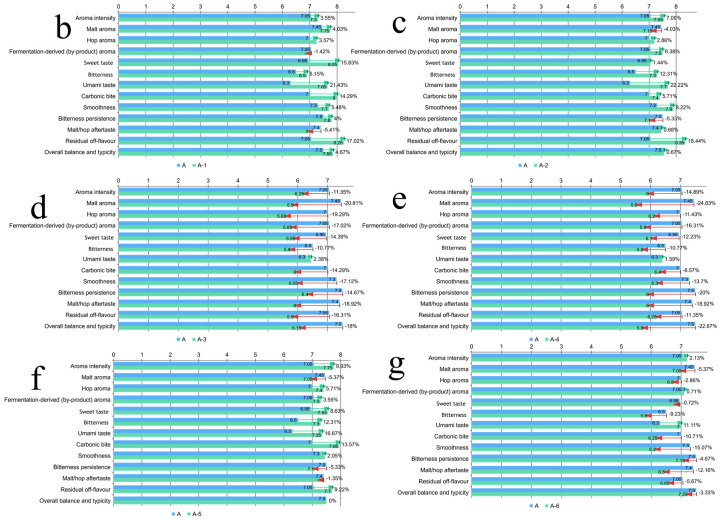
Multidimensional effects of the single-factor addition experiments on the beer-body sensory attributes. (**a**) The overall sensory scores of the beer samples. (**b**) The beer-body sensory attributes of the KSTEL-enriched sample were compared with those of the original beer. (**c**) The beer-body sensory attributes of the DELIK-enriched sample were compared with those of the original beer. (**d**) The beer-body sensory attributes of the DIGISSK-enriched sample were compared with those of the original beer. (**e**) The beer-body sensory attributes of the IEKYSGA-enriched sample were compared with those of the original beer. (**f**) The beer-body sensory attributes of the DEVR-enriched sample were compared with those of the original beer. (**g**) The beer-body sensory attributes of the PVPL-enriched sample were compared with those of the original beer.

As illustrated in Figure 6a and Table A2, increases in umami sensory scores were observed in every sample after single-peptide addition, and the magnitudes were systematically associated with binding free energies and taste thresholds. For DELIK (A-2), an umami score of 7.7 + 1.78 was recorded, about 22% higher than that of sample A (*p* < 0.05). The lowest binding free energy (−44.09 kcal mol^−1^) was also calculated, indicating the strongest affinity for the T1R1/T1R3 receptor. For DEVR (A-5), a comparable free energy (−43.21 kcal mol^−1^) and a low threshold (0.121 mmol L^−1^) were measured; consequently, the highest per-unit sensory efficiency was achieved and the score reached 7.35 + 1.6 (*p* < 0.05). KSTEL (A-1) yielded a high score (7.65 + 1.9) and a low threshold (0.217 mmol L^−1^), confirming pronounced enhancement (*p* < 0.05). PVPL (A-6) showed moderate values: a score of 7 + 2.71, a free energy of −39.53 kcal mol^−1^, and a relatively high threshold (0.589 mmol L^−1^), suggesting limited efficiency. For IEKYSGA (A-4), the umami score was increased by under 2%. A moderately negative binding free energy (−39.60 kcal mol^−1^) was recorded, but an unfavorable taste threshold offset this advantage, so only a slight net benefit was observed. DIGISSK (A-3) performed the worst: the score reached only 6.45 + 1.67 (*p* < 0.05), the least negative free energy (−26.45 kcal mol^−1^) was obtained, and the highest threshold (0.696 mmol L^−1^) was recorded, reflecting weak affinity and minimal efficiency.

The structural analysis revealed shared residue patterns among the top-performing peptides—DELIK, DEVR, and KSTEL. Acidic residues, such as Asp and Glu, were enriched at the N-terminus and were able to form stable electrostatic interactions with positively charged sites in the receptor binding domain. Basic or hydrophobic residues, such as Lys, Arg, Leu, and Val, were located at the C-terminus and further stabilized the complex through hydrogen bonding or hydrophobic contacts. The chain lengths were confined to four or five residues, thereby reducing conformational-entropy penalties and facilitating insertion into the receptor pocket. These features collectively resulted in lower binding free energies, higher sensory scores, and favorable thresholds, providing clear guidance for the rational design of future umami peptides.

#### 3.5.2. Multidimensional Effects of Single Umami Peptide Addition on Beer-Body Sensory Attributes

As Figure 6a indicates, an overall sensory improvement was observed for sample A-1 in comparison with sample A. Aroma intensity, malt aroma, and hop aroma were raised by about 3–4%, whereas fermentation-by-product aroma was reduced by roughly 1%. Sweetness and umami were enhanced by approximately 15% and 20%, and bitterness was lifted by about 6%, giving a more layered taste. Carbonic bite increased by nearly 14%, and smoothness also rose by close to 6%. Bitter aftertaste and overall balance were improved by around 4–5%. Malt/hop after-flavor fell by about 5%, while off-flavors climbed by roughly 17%.

In Figure 6c, comprehensive enhancement with local attenuation was recorded for sample A-2. Aroma intensity rose by about 7%. Hop aroma was lifted by nearly 3%, but malt aroma dropped by roughly 4%, indicating a slight masking of malt notes by the hops. Fermentation-by-product aroma climbed by about 6%. Umami increased the most (≈22%); bitterness grew by ≈12%; and sweetness changed little, rising by only about 1%. Carbonic bite and smoothness were raised by roughly 6% and 8%, respectively, creating a brisker and finer mouthfeel. Bitter aftertaste declined by about 5%. Off-flavors expanded by nearly 18%. Overall balance and typicality rose by less than 1%.

As shown in Figure 6d, most sensory attributes declined for sample A-3. Aroma parameters dropped by more than 10%, with malt aroma down by almost 20%, and hop aroma and fermentation-by-product aroma lower by about 20% and 17%. Sweetness and bitterness fell by roughly 15% and 10%. Carbonic bite and smoothness each decreased by over 10%. Malt/hop after-flavor shortened by nearly 20%, bitter aftertaste lessened by about 15%, and overall balance decreased by close to 20%. Umami rose by roughly 2%, and off-flavors fell by about 16%. The DIGISSK peptide, therefore, slightly intensified umami but produced marked negative effects on aroma fullness, mouthfeel smoothness, and flavor harmony.

Figure 6e demonstrated that almost all thirteen indicators declined for sample A-4. Malt aroma and fermentation-by-product aroma dropped by about 25% and 16%, while aroma intensity and hop aroma decreased by roughly 15% and 11%. Sweetness and bitterness were reduced by about 12% and 11%. Umami was the sole positive parameter, but the rise was only around 2%, insufficient to offset the overall flavor loss. Carbonic bite and smoothness fell by roughly 9% and 14%. Bitter aftertaste and malt/hop after-flavor were shortened by about 20% and 19%. Off-flavors fell by roughly 11%. Overall balance and typicality declined by about 22%, indicating that this peptide weakened aroma richness and flavor harmony under the present formulation.

For sample A-5 (Figure 6f), aroma intensity increased by about 10%, hop aroma rose by nearly 6%, and malt aroma fell by roughly 5%. Umami was enhanced by about 17%, and bitterness and sweetness climbed by approximately 12% and 9%. Carbonic bite was elevated by around 14% and smoothness by about 2%. Bitter aftertaste and malt/hop after-flavor decreased by roughly 5% and 1%. Off-flavors gained about 9%. Overall balance remained unchanged.

As Figure 6g shows, aroma intensity for sample A-6 increased by about 2.1%. Malt aroma and hop aroma dropped by roughly 5.4% and 2.9%, while fermentation-by-product aroma rose by about 0.7%. Umami was elevated by about 11.1%. Sweetness shifted by −0.7%, and bitterness declined by about 9.2%. The most pronounced negative effects occurred in mouthfeel, where carbonic bite and smoothness decreased by roughly 10.7% and 15.1%. Bitter aftertaste and malt/hop after-flavor were lowered by about 4.7% and 12.2%. Off-flavors fell by roughly 5.7%, suggesting a partial masking of negative notes. Overall balance and typicality declined by about 3.3%, showing that enhanced umami did not compensate for weakened aroma and mouthfeel.

A cross-comparison of the three datasets confirmed that computational predictions, threshold measurements, and sensory outcomes were largely coherent. The peptides showing the most negative binding free energies—DELIK, DEVR, and KSTEL—also exhibited the lowest taste thresholds and delivered the highest increases in umami, sweetness, and mouthfeel during sensory validation, supporting the reliability of the molecular-modeling workflow. DIGISSK and IEKYSGA, whose free energies were least negative and thresholds highest, provided only marginal sensory gains, again matching expectations. Minor inconsistencies, notably the moderate sensory impact of PVPL despite a mid-range free energy, were attributed to complex matrix interactions in beer, where synergistic or masking effects among volatiles, polyphenols, and carbonation could attenuate the direct contribution of a single peptide. Overall, single additions of short-chain umami peptides strengthened the “umami–sweetness–mouthfeel” framework, but the same additions tended to elevate off-flavors. Future optimization should therefore retain peptides with favorable computed affinity and low thresholds while adjusting fermentation and antioxidant conditions to limit by-product formation, thereby delivering a balanced, umami-oriented lager profile.

### 3.6. Discussion

Comparison with previously reported umami peptides highlights the exceptional potency of the sequences isolated here. Classical di- and tripeptides, such as Glu-Asp, Ala-Glu-Ala, and EY, exhibited taste threshold ranges of 0.5–2.2 mmol L^−1^, while the beefy meaty octapeptide Lys-Gly-Asp-Glu-Glu-Ser-Leu-Ala, long regarded as a benchmark, showed a threshold around 0.8 mmol L^−1^ [69,70,71]. By contrast, the beer-derived tetrapeptide DEVR (Asp-Glu-Val-Arg) and pentapeptide KSTEL (Lys-Ser-Thr-Glu-Leu) recorded markedly lower thresholds of 0.121 and 0.217 mmol L^−1^, respectively, and exhibited stronger receptor affinities (ΔG_bind_ ≤ −44 kcal mol^−1^) than the −31 kcal mol^−1^ reported for glutamyl dipeptides in silico [72]. A sequence motif analysis further shows that, consistent with the acidic “XXE/EDX” signatures common to known umami peptides, all the top-performing beer peptides contained terminal Asp or Glu residues that anchor within the T1R1 SB pocket. Collectively, these data position DEVR and KSTEL among the most potent umami peptides reported to date and confirm that malting and fermentation-driven proteolysis can generate highly active taste molecules in lager beer.

Sequence mapping against the barley proteome showed that our most potent peptides—for example, DEVR and KSTEL—display ≥ 80% identity to internal motifs of B and γ hordeins, while PVPL aligns with a C-terminal fragment of lipid transfer protein 1 [73]. Such acidic, Lys/Arg adjacent cleavage patterns are characteristic of endoprotease B and cathepsin-like enzymes that become highly active during germination and hot mashing, releasing hundreds of short peptides into wort. Subsequent yeast autolysis and secretion of vacuolar proteinase A (Pep4p) during late fermentation and conditioning further truncates these fragments to the 4–6 residue length optimal for umami activity. Collectively, these malt- and yeast-driven proteolytic events provide a plausible biosynthetic route for the generation of highly active umami peptides in lager beer, consistent with recent petidomic surveys that have detected comparable hordein-derived sequences across diverse beer styles [73,74,75].

Several limitations warrant consideration before the conclusions are drawn. First, the peptide identification and affinity predictions were based on pilot-scale brews and in silico docking to the T1R1/T1R3 ectodomain. The potential matrix effects, post-packaging degradation, and contributions from other taste receptors (e.g., mGluR4) were not explored. Second, the sensory validation relied on a relatively small, trained panel, which—although sufficient for discriminative testing—may not capture broader consumer heterogeneity. Third, this study focused on a single pale-lager recipe brewed under controlled laboratory conditions. The results may differ across malt varieties, hop schedules, or commercial production environments. Finally, the single-addition design did not investigate synergistic or antagonistic interactions among peptides and endogenous beer components. These constraints highlight the need for follow-up work encompassing larger consumer datasets, multiple beer matrices, and comprehensive receptor profiling to confirm the generality and practical applicability of the present findings.

## 4. Conclusions

LC-MS/MS combined with de novo sequencing and database searching was first employed to identify 906 peptides in lager beer, and 76 potential umami peptides were predicted with UMPred-FRL, TastePeptides-Meta, and Umami-MRNN. Integrated molecular docking, molecular-dynamics simulation, and MM/GBSA calculations were then used to select six representative umami peptides—KSTEL, DELIK, DIGISSK, IEKYSGA, DEVR, and PVPL. DEVR, KSTEL, and DELIK showed the lowest binding free energies (ΔG_bind_ ≈ −44.09, −43.21, and −36.14 kcal mol^−1^) and formed compact hydrogen-bond networks in the T1R1/T1R3 interface, indicating the strongest receptor affinity and conformational stability, whereas DIGISSK bound most weakly and remained the most flexible.

Computational screening identified short-chain peptides rich in Asp/Glu at the N-terminus and Lys/Arg or hydrophobic residues at the C-terminus as the most promising ligands. Their low binding free energies (≈−44 to −36 kcal mol^−1^) coincided with sub-millimolar taste thresholds (≈0.12–0.40 mmol L^−1^) and the largest sensory gains, confirming the efficiency and accuracy of the in silico workflow. Sensory validation showed that these peptides strengthened the “umami–sweetness–mouthfeel” dimension, whereas peptides with weaker affinities and higher thresholds produced minimal or negative effects. The matrix interactions in beer occasionally dampened the expected impact, highlighting that, although calculation-guided selection accelerated discovery, formulation optimization remained necessary to balance flavor enhancement against potential off-flavors.

In conclusion, KSTEL, DELIK, and DEVR emerged as core umami peptides for an “umami-oriented” lager, combining high affinity, low thresholds, and pronounced sensory enhancement. Because umami reinforcement correlated positively with off-flavors, fermentation and antioxidant strategies should be optimized to suppress by-products and oxidation products, thereby achieving both umami enhancement and flavor harmony. The present “structure–receptor–sensory” workflow supplied clear guidance and key parameters for designing high-quality umami peptides and applying them in beer.

## Figures and Tables

**Table 1:** Liquid chromatography separation conditions.

Time (min)	Flow Velocity (mL/min)	A%	B%
0	0.2	95	5
0.5	0.2	95	5
24	0.2	30	70
24.1	0.2	10	90
27	0.2	10	90
27.1	0.2	95	5
30	0.2	95	5

**Table 2:** Mass spectrometer ion source parameters.

Mass Spectrometry Ion Source Parameters	Set Value
Spray voltage	4.0 kV
Sheath gas flow rate	35 °C
Auxiliary gas flow rate	15 mL/min
Capillary temperature	300 °C
S-lens RF power	30 eV

**Table 3:** Sensory dimensions description and scoring.

Sensory Dimension	Sensory Description	Rating
Aroma intensity	The predominant aroma is malt freshness, with weaker hop and byproduct odors, resulting in an overall clean and pure flavor profile, with no off-tastes.	0 (No aroma)–9 (Very strong aroma)
Malt aroma	The fragrance includes fresh bread and light caramel sweetness from the malt, with no burnt or harsh aftertastes.	0 (No malt aroma)–9 (Extremely strong malt aroma)
Hop aroma	Herbal floral or light citrus notes provide a refreshing and complementary aroma, without any sharp or oxidized odors.	0 (No hops aroma)–9 (Extremely strong hops aroma)
Fermentation-derived (by-product) aroma	Low ester fruitiness or light sulfur notes are present, maintaining the “clean” characteristic of lagers, with no phenolic flavors.	0 (Strong by-product flavors that affect sensory perception of the body)–9 (Balanced by-product aroma)
Sweet taste	Malt sweetness is accompanied by a hint of honey and biscuit flavors, finishing cleanly.	0 (No malt flavor)–9 (Extremely strong malt flavor)
Bitterness	The herbal or floral bitterness is smooth and balanced with malt sweetness, without any harshness.	0 (No hops flavor)–9 (Extremely strong hops flavor)
Umami taste	Free amino acids and peptides contribute to a gentle aftertaste and full-bodied sensation, without any umami or MSG-like flavors.	0 (No fresh flavor)–9 (Extremely strong fresh flavor)
Carbonic bite	The crisp and stimulating sensation from carbonation is felt as a tingling on the tip of the tongue and a refreshing throat feel.	0 (Completely flat)–9 (Extremely stimulating)
Smoothness	The mouthfeel is smooth and refined, with no rough or harsh textures.	0 (Very rough)–9 (Extremely smooth)
Bitterness persistence	Post-swallow bitterness is short-lived and refreshing, without any sharpness.	0 (No bitterness)–9 (Extremely strong bitterness)
Malt/hop aftertaste	A lingering malt sweetness and floral/herbal aftertaste provide a brief but pleasant finish.	0 (No aftertaste)–9 (Rich and lasting aftertaste)
Residual off-flavor	No sour, phenolic, metallic, or cardboard-like off-flavors are present.	0 (Heavy aftertaste of defects)–9 (Long aftertaste)
Overall balance and typicity	Malt, hop, freshness, and carbonation are well-balanced, characteristic of a typical pale lager.	0 (Unbalanced and atypical)–9 (Perfectly balanced and highly typical)

**Table 4:** Comparison of binding energy of potential and reported umami peptides with T1R1/T1R3 receptor.

Number	Peptide Sequence	Peptide Chain Length	ΔE_docking_ (kcal/mol)	ΔE_interaction_ (kcal/mol)	ΔE_binding_ (kcal/mol)
1	DIGISSK	7	−125.028	−107.79	−238.433
2	IEKYSGA	7	−123.489	−105.566	−236.545
3	AAEVIE	6	−115.817	−84.9475	−240.514
4	KSTEL	5	−111.033	−108.351	−501.245
5	AASEGKL	7	−110.848	−102.666	−221.33
6	KVGADK	6	−108.145	−89.6395	−241.495
7	KEELE	5	−107.783	−88.7778	−314.491
8	DVVAI	5	−107.637	−87.5399	−175.456
9	QELQLQ	6	−106.492	−91.267	−149.841
10	DEVR	4	−105.523	−76.4819	−186.616
11	FATPLQ	6	−103.521	−102.69	−326.679
12	DELIK	5	−102.52	−81.7171	−254.719
13	EAAVL	5	−102.002	−81.779	−201.669
14	VEILN	5	−101.716	−96.1308	−282.968
15	DELR	4	−100.336	−88.69	−361.423
16	EVGAL	5	−99.6013	−83.949	−218.184
17	LGGVE	5	−97.9888	−77.7048	−260.41
18	AAEVI	5	−97.3921	−65.5859	−62.5942
19	IAAVE	5	−96.4886	−84.0782	−301.133
20	IGTPGKG	7	−95.8082	−104.236	−333.345
21	VDAGI	5	−94.9971	−80.5047	−191.284
22	TIADV	5	−94.5377	−72.3421	−149.121
23	LGAVD	5	−93.5717	−82.6503	−280.053
24	LAGVE	5	−91.5818	−62.7922	−123.894
25	IGAVD	5	−90.8998	−68.4441	−166.599
26	AAGQY	5	−90.548	−82.4856	−311.285
27	AAEVL	5	−89.9893	−70.3364	−208.152
28	VSVVD	5	−89.9392	−79.0146	−255.595
29	LAAVE	5	−89.1563	−79.0668	−254.727
30	TAEPY	5	−84.7037	−85.4494	−206.263
31	TVSGF	5	−84.16	−73.4952	−171.631
32	TTVSPH	6	−83.9876	−100.04	−365.475
33	KNCQLA	6	−83.945	−82.4215	−222.162
34	TVVSA	5	−82.9071	−76.1503	−204.041
35	IVMQQ	5	−82.6036	−81.4959	−225.551
36	TATVP	5	−77.9485	−79.7372	−298.009
37	TVTVP	5	−77.2092	−87.853	−334.745
38	LPEDA	5	−75.7012	−80.2355	−170.975
39	VLQDR	5	−75.5022	−69.5075	−155.017
40	TVATP	5	−73.8591	−78.7827	−269.535
41	TLPLT	5	−73.7163	−75.0438	−133.258
42	TTVSP	5	−73.4146	−74.2375	−184.347
43	TVTSP	5	−71.5777	−78.5044	−240.692
44	KRTP	4	−70.9232	−85.6676	−294.082
45	LPSLQ	5	−69.9013	−75.7618	−175.372
46	LDLP	4	−69.893	−66.5489	−150.146
47	PVAPLQ	6	−69.867	−87.1519	−260.307
48	TNLP	4	−66.6257	−74.2949	−286.005
49	AVAYDP	6	−65.1639	−71.822	−67.7682
50	LPSNP	5	−61.5173	−73.2008	−231.768
51	PSPNN	5	−58.9083	−77.5908	−230.304
52	AAVLEY	6	−58.487	−62.9721	786.893
53	TVSP	4	−57.4176	−63.4953	−208.571
54	LPTKP	5	−56.7889	−80.1074	−256.268
55	VEVMR	5	−54.8233	−60.8928	−31.4658
56	AIVMQQ	6	−47.7704	−78.7023	−336.775
57	TLPQQP	6	−41.7705	−71.3837	−162.521
56	* PVPL	4	−39.6184	−57.8106	−65.824

Note: *: reported umami peptides [16], ΔE_docking_: docking energy, ΔE_interaction_: interaction energy, ΔE_binding_: binding energy.

**Table 5:** Basic information, taste description, and threshold of selected umami peptides.

Peptide Sequence	Peptide Chain Length	Mass	Peptide Source	Taste Description	Umami Threshold (mmol/L)
KSTEL	5	576.312	Triticum turgidum, barley	Typical umami with a slight hint of saltiness	0.217
DELIK	5	616.343	Triticum turgidum, barley	Umami-salty composite, virtually no bitterness	0.406
DIGISSK	7	718.386	Saccharomyces cerevisiae, barley	Umami with a bready/yeast-like aftertaste	0.696
IEKYSGA	7	766.386	Saccharomyces cerevisiae	Umami accompanied by a mild sweetness	0.326
DEVR	4	517.250	Triticum turgidum, barley	Umami with a subtle salty note	0.121
PVPL	4	424.540	Oryza, wild rice, durum wheat	Mild umami with a touch of sweetness	0.589

**Table 6:** Binding free energies and energy components predicted by MM/GBSA (kcal/mol).

System	ΔE_vdW_	ΔE_elec_	ΔG_GB_	ΔG_SA_	ΔG_bind_
T1R1-T1R3/DELIK	−52.83 ± 3.39	−177.59 ± 17.87	202.53 ± 16.15	−8.24 ± 0.24	−36.14 ± 3.11
T1R1-T1R3/DEVR	−42.78 ± 3.60	−190.13 ± 26.12	195.58 ± 18.95	−6.76 ± 0.32	−44.09 ± 5.47
T1R1-T1R3/DIGISSK	−47.66 ± 4.72	−156.25 ± 41.90	184.41 ± 37.34	−6.96 ± 0.64	−26.45 ± 4.52
T1R1-T1R3/IEKYSGA	−54.27 ± 2.13	−196.15 ± 23.49	220.33 ± 20.81	−9.51 ± 0.22	−39.60 ± 4.37
T1R1-T1R3/KSTEL	−52.77 ± 3.08	−244.96 ± 18.17	262.45 ± 17.99	−7.93 ± 0.20	−43.21 ± 3.45
T1R1-T1R3/PVPL	−45.93 ± 2.03	−42.53 ± 11.04	55.49 ± 10.81	−6.51 ± 0.39	−39.53 ± 2.52

Note: ΔE_vdW_: van der Waals energy; ΔE_elec_: electrostatic energy; ΔG_GB_: electrostatic contribution to solvation; ΔG_SA_: non-polar contribution to solvation; ΔG_bind_: binding free energy.

## Data Availability

The original contributions presented in the study are included in the article, further inquiries can be directed to the corresponding author.

## Appendix A

**Table A1:** Qualitative parameters of peptides in lager beer and predicted umami values.

Number	Peptide Chain	−10lgP	Mass	*m*/*z*	RT	ALC (%)	Class	UMPred-FRL-Probability	ProUmami
1	VEILN	15.200	586.333	587.342	8.880		Umami	0.992	0.98
2	AAEVLE	15.660	630.322	631.331	6.800		Umami	0.988	0.98
3	IGAVD	16.980	473.249	474.257	5.630		Umami	0.982	0.989
4	KEELE	16.740	646.317	647.326	2.740		Umami	0.98	0.977
5	TATVP	17.220	487.264	488.273	6.020		Umami	0.979	0.983
6	LVAP		398.253	399.261	7.260	98	Umami	0.977	0.008
7	IEKYSGA	16.020	766.386	384.201	3.420		Umami	0.977	0.977
8	EGAVP	15.890	471.233	472.242	5.070		Umami	0.977	0.968
9	EAAVI	15.270	501.280	502.289	7.440		Umami	0.975	0.939
10	AAVLEY	17.470	664.343	665.353	8.730		Umami	0.975	0.983
11	IAAVE	16.450	501.280	502.288	7.590		Umami	0.974	0.981
12	VEVMR		632.332	317.174	5.090	98	Umami	0.973	0.89
13	NLFDVNRP	18.930	973.498	487.758	8.430		Umami	0.972	0.984
14	TPLQP	15.560	554.306	555.315	6.800	94	Umami	0.97	0.253
15	LAGVE	16.220	487.264	488.273	6.560		Umami	0.969	0.983
16	AFTPLQ		675.359	676.369	8.710	96	Umami	0.968	0.992
17	FATPLQ		675.359	676.369	8.800	97	Umami	0.966	0.991
18	TTVSPH	16.900	640.318	641.328	2.510		Umami	0.962	0.982
19	TVTVP	15.090	515.296	516.305	7.620		Umami	0.961	0.974
20	PVAPLQ		623.364	624.373	7.580	91	Umami	0.961	0.982
21	LGAVD	16.470	473.249	474.257	5.630		Umami	0.961	0.987
22	EGGVL	16.700	473.249	474.257	7.180		Umami	0.961	0.969
23	TAAVV	16.140	459.269	460.277	3.040		Umami	0.96	0.305
24	AAEVIE	15.660	630.322	631.331	6.800		Umami	0.96	0.981
25	TAEPY		579.254	580.263	5.270	90	Umami	0.959	0.979
26	VPMP		442.225	443.234	7.860	97	Umami	0.955	0.011
27	LGGVE	16.110	473.249	474.258	5.830		Umami	0.953	0.982
28	FAVP		432.237	433.246	9.200	96	Umami	0.953	0.021
29	TLPLT	18.050	543.327	544.336	8.960		Umami	0.949	0.974
30	TVSGF	18.850	509.249	510.257	7.120	94	Umami	0.945	0.985
31	LSVGI	15.230	487.301	488.310	8.990		Umami	0.943	0.08
32	TTVSP	20.430	503.259	504.267	4.460	90	Umami	0.94	0.981
33	AAEVI	15.190	501.280	502.289	7.440		Umami	0.938	0.985
34	MFADHLA	15.440	803.364	804.366	2.850		Umami	0.936	0.978
35	DVAGI	15.070	473.249	474.257	6.990		Umami	0.934	0.984
36	ERLP		513.291	514.299	4.540	95	Umami	0.932	0.881
37	TVQVELTTEK		1147.597	574.808	7.570	91	Umami	0.931	0.978
38	LDLP		456.258	457.267	7.920	90	Umami	0.929	0.731
39	VPVP		410.253	411.261	7.520	98	Umami	0.928	0.01
40	LVTGGDSGIGRA	15.250	1101.578	551.798	6.450		Umami	0.928	0.978
41	LPSLQ	15.170	556.322	557.331	8.160	95	Umami	0.927	0.975
42	AAADDEEMKL	23.340	1091.481	546.749	7.380	93	Umami	0.925	0.978
43	DVAGL	15.070	473.249	474.257	6.990		Umami	0.918	0.985
44	LVGL		400.269	401.277	9.510	98	Umami	0.915	0.008
45	TVTSP	18.670	503.259	504.269	4.940	90	Umami	0.906	0.98
46	VDAGI	15.250	473.249	474.257	6.990		Umami	0.903	0.987
47	LAAVE	16.450	501.280	502.288	7.590		Umami	0.902	0.983
48	DEVR		517.250	518.259	2.510	91	Umami	0.898	0.978
49	VVLPSTE	21.060	743.407	744.417	7.960	95	Umami	0.889	0.977
50	LEKYSGA		766.386	384.201	3.420	97	Umami	0.889	0.975
51	AAGQY		508.228	509.237	2.870	93	Umami	0.887	0.974
52	EAAVL	15.270	501.280	502.289	7.440		Umami	0.886	0.966
53	WFRSHT	15.130	832.398	833.397	7.800		Umami	0.884	0.922
54	LVALP	16.500	511.337	512.347	11.790	98	Umami	0.883	0.033
55	FVTP		462.248	463.257	7.820	97	Umami	0.883	0.122
56	TVSP		402.211	403.220	3.780	92	Umami	0.881	0.928
57	KNCQLA	17.540	675.337	676.344	6.300		Umami	0.881	0.987
58	FVRLL		646.417	324.216	9.530	97	Umami	0.88	0.017
59	DVVAI	15.060	515.296	516.305	8.020		Umami	0.88	0.983
60	GDKLIVHA	17.200	851.487	852.496	7.690		Umami	0.879	0.936
61	LPEDA		543.254	544.262	5.550	98	Umami	0.877	0.976
62	LPSNP	17.960	526.275	527.283	5.590	99	Umami	0.875	0.982
63	FVDVVP	17.090	674.364	675.375	11.810		Umami	0.875	0.987
64	PPPVHDTD	25.290	876.398	439.208	3.360		Umami	0.873	0.965
65	LSVGL	15.230	487.301	488.310	8.990		Umami	0.867	0.586
66	ISVGL	15.230	487.301	488.310	8.990		Umami	0.865	0.059
67	KVGADK		658.365	659.374	2.910	94	Umami	0.862	0.988
68	IVATPLL	18.490	725.469	726.479	11.950		Umami	0.862	0.965
69	VPAP		382.222	383.229	5.610	99	Umami	0.861	0.017
70	TTGGMRPP	18.370	815.396	408.706	5.500	95	Umami	0.849	0.974
71	KRTP		542.318	543.328	2.510	96	Umami	0.849	0.894
72	LSLAL	16.100	515.332	516.341	10.360		Umami	0.848	0.314
73	LALSL	15.390	515.332	516.341	11.090	92	Umami	0.848	0.917
74	AAEVL	15.190	501.280	502.289	7.440		Umami	0.843	0.984
75	AAKMAK		660.363	331.190	3.040	97	Umami	0.843	0.094
76	AGEQAFHRG	25.320	1013.468	507.742	5.610		Umami	0.842	0.986
77	LPTKP		554.343	555.351	4.390	93	Umami	0.837	0.843
78	DELR		531.265	532.275	3.920	96	Umami	0.835	0.978
79	KVVVP		540.364	541.372	6.900	92	Umami	0.825	0.014
80	DIGISSKA	19.120	789.423	790.434	6.420		Umami	0.825	0.978
81	LAAP		370.222	371.230	5.910	94	Umami	0.814	0.04
82	LSGAL	18.080	459.269	460.279	9.310		Umami	0.81	0.275
83	VLTSNVGANR	15.940	1030.541	516.280	6.110		Umami	0.806	0.971
84	AIVMQQ	16.760	688.358	689.367	6.890		Umami	0.806	0.984
85	IGTPGKG	17.770	628.354	315.185	2.860		Umami	0.805	0.973
86	VLQDR		629.350	315.684	2.700	90	Umami	0.801	0.98
87	TLPIT	16.980	543.327	544.336	8.960		Umami	0.8	0.959
88	DELIK	16.300	616.343	617.352	6.020		Umami	0.797	0.977
89	TPLP		426.248	427.256	7.430	98	Umami	0.796	0.057
90	TVATP	15.370	487.264	488.272	5.240		Umami	0.792	0.978
91	AGEQAFH	21.710	800.345	801.355	6.600		Umami	0.791	0.988
92	ALLSL	19.520	515.332	516.341	11.090		Umami	0.79	0.142
93	VSVVD	17.390	517.275	518.284	6.220		Umami	0.785	0.984
94	QQPLP	17.730	581.317	582.327	7.000		Umami	0.785	0.074
95	AASEGKL	25.540	674.360	675.369	3.100		Umami	0.777	0.979
96	AVAYDP	15.340	634.296	635.305	6.010		Umami	0.772	0.983
97	KSTEL		576.312	577.321	3.080	92	Umami	0.771	0.978
98	TVVSA	19.430	475.264	476.273	6.510		Umami	0.765	0.967
99	IVTGGDSGIGRA	16.000	1101.578	551.798	6.450		Umami	0.763	0.978
100	FSVF		498.248	499.256	11.110	97	Umami	0.761	0.174
101	GVQMK	15.230	561.294	562.304	2.710		Umami	0.76	0.582
102	VGLP		384.237	385.246	8.220	93	Umami	0.759	0.197
103	DIGISSK	18.030	718.386	719.397	5.910		Umami	0.755	0.978
104	NFVLR		648.360	649.369	9.310	97	Umami	0.752	0.044
105	VDVSVVD	17.810	731.370	732.380	8.150		Umami	0.751	0.976
106	LALRTLP		782.501	392.259	8.920	92	Umami	0.747	0.981
107	QELQLQ	16.270	757.397	758.408	6.170		Umami	0.745	0.98
108	PSPNN	17.800	527.234	528.242	1.100		Umami	0.745	0.982
109	AIVMQQQ	16.720	816.416	817.425	6.640		Umami	0.739	0.971
110	VATLFPL	16.330	759.453	760.462	13.980		Umami	0.736	0.963
111	TNLP		443.238	444.247	6.320	93	Umami	0.734	0.968
112	MPLE		488.231	489.238	7.230	99	Umami	0.734	0.546
113	TIADV	15.910	517.275	518.284	6.320		Umami	0.73	0.982
114	ERFQPM	19.200	822.369	412.194	4.990		Umami	0.729	0.965
115	LLVP		440.300	441.308	9.640	95	Umami	0.725	0.009
116	LTLP		442.279	443.288	9.600	94	Umami	0.723	0.007
117	EVGAL	17.250	487.264	488.272	7.210		Umami	0.717	0.984
118	TLPQQP		682.365	683.375	6.230	90	Umami	0.714	0.97
119	IVMQQ	15.400	617.321	618.331	6.010		Umami	0.71	0.987
120	QELQIQ	17.280	757.397	758.408	6.170		Umami	0.701	0.979
121	VGSAI	19.110	445.254	446.261	6.730		Umami	0.7	0.597
122	SLVLRTLP	17.260	897.565	449.791	10.030		Umami	0.698	0.977
123	TPVF		462.248	463.257	8.630	91	Umami	0.697	0.147
124	VAGLP	16.130	455.274	456.283	8.330		Umami	0.695	0.358
125	SLVGI	20.410	487.301	488.310	9.070		Umami	0.693	0.015
126	ANLPDR		684.356	343.186	4.080	95	Umami	0.689	0.984
127	LVMQQ	16.160	617.321	618.331	6.010	93	Umami	0.686	0.984
128	LFSVP	15.940	561.316	562.326	10.150		Umami	0.678	0.51
129	TLSTM		567.257	568.266	3.670	92	Umami	0.671	0.978
130	LEAVP	15.980	527.296	528.305	8.600	98	Umami	0.666	0.971
131	GSLLL	18.680	501.316	502.325	11.050		Umami	0.664	0.038
132	ALAR		429.270	430.279	2.660	96	Umami	0.663	0.046
133	TVSSVP	15.600	588.312	589.321	7.500		Umami	0.659	0.981
134	LGALSG	16.460	516.291	517.300	6.540		Umami	0.659	0.966
135	DTVR		489.255	490.266	2.140	94	Umami	0.655	0.981
136	LPEW		543.269	544.278	9.850	98	Umami	0.65	0.047
137	KGAP		371.217	372.225	1.770	98	Umami	0.648	0.077
138	DEILK	16.300	616.343	617.352	6.020		Umami	0.641	0.977
139	KSSL		475.264	476.274	7.980	95	Umami	0.639	0.988
140	AILSL	19.520	515.332	516.341	11.090		Umami	0.637	0.039
141	KPSVYP	21.150	689.375	690.386	6.050		Umami	0.636	0.268
142	SIALRTLP	21.060	869.533	870.544	9.240		Umami	0.635	0.979
143	LSLP		428.264	429.272	9.420	96	Umami	0.635	0.06
144	LLVTP	15.510	541.348	542.357	9.160		Umami	0.626	0.013
145	QHIAQLE	16.950	837.434	419.726	6.330		Umami	0.62	0.981
146	LVLP		440.300	441.309	10.400	94	Umami	0.616	0.01
147	TVASP	15.000	473.249	474.257	5.740		Umami	0.611	0.97
148	LSIAL	16.100	515.332	516.341	10.360		Umami	0.605	0.042
149	INGNK	16.040	544.297	545.307	2.970		Umami	0.605	0.99
150	GLSSL	18.870	475.264	476.274	7.880		Umami	0.599	0.985
151	AGEQAFHRGG	25.390	1070.489	536.253	5.550		Umami	0.599	0.985
152	RTTPVG	17.590	629.350	630.358	3.120		Umami	0.596	0.974
153	GASLI	20.160	459.269	460.279	8.090		Umami	0.593	0.576
154	VGGH		368.181	369.188	1.800	95	Umami	0.583	0.06
155	LPVD		442.243	443.252	6.520	90	Umami	0.57	0.975
156	ERFQP	16.230	675.334	676.344	5.420		Umami	0.566	0.983
157	VLFSVP	16.580	660.385	661.394	10.950	90	Umami	0.561	0.914
158	QQQLP	15.700	612.323	613.334	6.150		Umami	0.556	0.984
159	IVATP	15.240	499.301	500.309	6.580		Umami	0.549	0.247
160	EAGAVRP	15.880	698.371	699.381	6.240		Umami	0.547	0.983
161	QLPHT		594.313	595.322	5.480	90	Umami	0.543	0.956
162	KSLVGY	18.370	665.375	666.382	4.500		Umami	0.539	0.897
163	GASLL	20.160	459.269	460.279	8.090		Umami	0.539	0.782
164	AADESTGTIGK	27.150	1048.504	525.262	3.770		Umami	0.538	0.978
165	ADESTGTIGK	19.200	977.467	489.742	3.400		Umami	0.534	0.978
166	DLER		531.265	532.275	2.600	95	Umami	0.531	0.976
167	LSLAI	16.100	515.332	516.341	10.360		Umami	0.527	0.024
168	LPQQ		484.265	485.273	3.650	95	Umami	0.527	0.971
169	NARLD		588.287	589.297	4.880	91	Umami	0.522	0.98
170	DELLK	16.300	616.343	617.352	6.020		Umami	0.522	0.977
171	TSLALRTLP		970.581	486.299	9.380	91	Umami	0.521	0.977
172	FPVG		418.222	419.231	7.950	90	Umami	0.519	0.019
173	TIPLT	18.050	543.327	544.336	8.960		Umami	0.517	0.917
174	KSSSG	19.230	464.223	465.231	1.540		Umami	0.514	0.986
175	KASTP	15.820	502.275	503.283	1.960		Umami	0.513	0.977
176	SLVGL	20.410	487.301	488.310	9.070		Umami	0.509	0.013
177	ANTARQAFQ	16.730	1005.499	503.758	4.200		Umami	0.508	0.981
178	QPLPQP	18.020	678.370	679.379	7.450		Non-umami	0.5	0.042
179	KTGF		493.254	494.263	7.710	95	Non-umami	0.5	0.191
180	ILQAA	15.480	514.312	515.320	6.450		Non-umami	0.5	0.016
181	ALLSI	19.520	515.332	516.341	11.090		Non-umami	0.5	0.146
182	GASIL	20.160	459.269	460.279	8.090		Non-umami	0.488	0.192
183	LPVP		424.269	425.277	8.920	98	Non-umami	0.486	0.01
184	TVLGA	15.410	459.269	460.278	6.970		Non-umami	0.483	0.259
185	IAHGGVLPNIN	30.170	1103.609	552.814	8.600		Non-umami	0.482	0.964
186	EVVR		501.291	502.300	2.470	96	Non-umami	0.482	0.865
187	KSTSP	16.420	518.270	519.278	1.990		Non-umami	0.481	0.979
188	DEIIK	16.300	616.343	617.352	6.020		Non-umami	0.478	0.975
189	ILVTP	15.510	541.348	542.357	9.160		Non-umami	0.476	0.022
190	VELLN	15.200	586.333	587.342	8.880		Non-umami	0.466	0.981
191	IVALP	16.500	511.337	512.347	11.790		Non-umami	0.463	0.041
192	AASEIGK	15.930	674.360	675.369	3.190		Non-umami	0.458	0.98
193	GSLIL	18.680	501.316	502.325	11.050		Non-umami	0.457	0.032
194	APALP	15.170	467.274	468.283	7.420		Non-umami	0.454	0.072
195	LPQQP	18.010	581.317	582.325	5.570		Non-umami	0.453	0.95
196	LALSI	15.390	515.332	516.341	11.090		Non-umami	0.453	0.122
197	TAALL	18.200	487.301	488.309	6.100		Non-umami	0.45	0.104
198	FTPQQP	20.770	716.349	717.356	7.140		Non-umami	0.449	0.981
199	LPVQP		552.327	553.335	7.330	91	Non-umami	0.447	0.059
200	GELAK	15.670	516.291	517.300	2.380		Non-umami	0.444	0.981
201	ADLPGVK	15.990	698.396	350.207	6.490	97	Non-umami	0.444	0.986
202	IVTGGDSGIGR	16.650	1030.541	516.280	6.110		Non-umami	0.439	0.979
203	VPLLQ	15.110	568.358	569.368	9.390	92	Non-umami	0.435	0.201
204	LPENA		542.270	543.279	5.200	98	Non-umami	0.429	0.981
205	TVGIL	16.800	501.316	502.324	10.950		Non-umami	0.428	0.061
206	LSGAI	18.080	459.269	460.279	9.310		Non-umami	0.425	0.077
207	KVGF		449.264	450.272	5.630	99	Non-umami	0.423	0.02
208	TPGKG	17.080	458.249	459.256	1.840	95	Non-umami	0.419	0.065
209	ASSLKVA	15.950	674.396	675.407	5.790		Non-umami	0.416	0.986
210	AHVF		472.243	473.251	5.530	94	Non-umami	0.415	0.018
211	AFEPIRS	15.120	818.429	410.223	6.940		Non-umami	0.415	0.983
212	LPRSGP	15.160	625.355	313.685	3.970	98	Non-umami	0.412	0.986
213	LGTF		436.232	437.242	8.060	93	Non-umami	0.412	0.359
214	ALISL	19.520	515.332	516.341	11.090		Non-umami	0.411	0.039
215	YPEQP		632.281	633.290	5.720	90	Non-umami	0.409	0.979
216	TSIALRTLP	30.640	970.581	486.299	9.380		Non-umami	0.405	0.977
217	ESTLHLVLR	15.260	1066.614	356.546	8.540		Non-umami	0.404	0.977
218	LASGAL	16.810	530.306	531.314	7.210		Non-umami	0.403	0.966
219	HQPQPQ	15.040	733.351	734.361	2.230		Non-umami	0.403	0.809
220	EVGAI	17.250	487.264	488.272	7.210		Non-umami	0.403	0.939
221	DVVR		487.275	488.285	2.740	92	Non-umami	0.402	0.982
222	TVVGL	15.200	487.301	488.310	8.920	93	Non-umami	0.4	0.06
223	LIVTP	15.510	541.348	542.357	9.160		Non-umami	0.39	0.008
224	KVLVTP	15.230	655.427	656.436	6.890	92	Non-umami	0.39	0.897
225	EKVVVLAG	15.530	813.496	814.506	10.010		Non-umami	0.385	0.964
226	QQPQIP	18.750	709.376	710.385	5.280		Non-umami	0.383	0.857
227	SIVGI	20.410	487.301	488.310	9.070		Non-umami	0.382	0.015
228	KNVQ		487.276	488.280	5.570	93	Non-umami	0.382	0.989
229	QALEVLR		827.487	414.752	7.410	91	Non-umami	0.381	0.982
230	QLPQQP	19.750	709.376	710.385	5.750		Non-umami	0.38	0.971
231	GLSAIQ	16.360	587.328	588.339	8.460		Non-umami	0.375	0.984
232	IASGAI	17.190	530.306	531.314	7.210		Non-umami	0.373	0.979
233	PTAYNTLLR		1047.571	524.796	8.040	97	Non-umami	0.371	0.977
234	SVAAV	16.450	445.254	446.262	5.770		Non-umami	0.366	0.253
235	AVGVE	15.700	473.249	474.258	5.830		Non-umami	0.362	0.982
236	TVATPVL	15.140	699.417	700.426	9.850		Non-umami	0.361	0.982
237	VVVP		412.269	413.278	7.830	99	Non-umami	0.359	0.01
238	QAGLK	17.160	515.307	516.316	2.590		Non-umami	0.359	0.1
239	SASLK	18.270	504.291	505.300	2.310	98	Non-umami	0.355	0.95
240	DESTGTIGK	18.780	906.429	454.223	3.570		Non-umami	0.355	0.978
241	LGGLSS	17.300	532.286	533.295	6.450		Non-umami	0.354	0.985
242	GLSSI	18.870	475.264	476.274	7.880		Non-umami	0.354	0.984
243	FPEAP	15.090	559.264	560.274	7.730	93	Non-umami	0.353	0.149
244	VAGL		358.222	359.230	6.920	97	Non-umami	0.352	0.071
245	TVAGL	15.430	459.269	460.278	7.740		Non-umami	0.352	0.915
246	VGSVG	15.840	417.222	418.230	1.760		Non-umami	0.351	0.848
247	QHASGR	15.680	654.320	655.327	1.510		Non-umami	0.345	0.981
248	ISLAL	16.100	515.332	516.341	10.360		Non-umami	0.344	0.036
249	TLAGL	20.990	473.285	474.293	7.160	93	Non-umami	0.343	0.856
250	SVGVS	15.550	447.233	448.239	1.820		Non-umami	0.342	0.984
251	PTAYNTILR	25.740	1047.571	524.796	8.040		Non-umami	0.339	0.974
252	ALGAL	17.130	443.274	444.283	7.600		Non-umami	0.339	0.1
253	QQQIP	15.700	612.323	613.334	6.150		Non-umami	0.337	0.966
254	PEYQP	15.280	632.281	633.289	5.620	92	Non-umami	0.337	0.985
255	AVATPVFL	20.510	816.475	817.484	12.540		Non-umami	0.336	0.99
256	GSLLI	18.680	501.316	502.325	11.050		Non-umami	0.319	0.05
257	VALSL	18.620	501.316	502.325	10.340		Non-umami	0.317	0.955
258	VGAASIP	19.260	613.344	614.353	7.930		Non-umami	0.315	0.988
259	QYPQQP	15.090	759.355	760.366	4.800		Non-umami	0.314	0.977
260	LSGAVGL	15.010	615.359	616.369	9.290		Non-umami	0.314	0.983
261	VGFP		418.222	419.231	8.700	96	Non-umami	0.312	0.027
262	LAVATPVF	17.000	816.475	817.485	11.950		Non-umami	0.312	0.989
263	TLALGP		570.338	571.347	8.070	94	Non-umami	0.309	0.31
264	TITSR	16.560	576.323	577.334	2.240		Non-umami	0.308	0.98
265	ITTSR	15.120	576.323	577.334	2.240		Non-umami	0.308	0.979
266	LAISL	15.390	515.332	516.341	11.090		Non-umami	0.306	0.136
267	GISSL	18.870	475.264	476.274	7.880		Non-umami	0.305	0.986
268	LTGMAFRVP	20.670	990.532	496.275	10.450	98	Non-umami	0.303	0.968
269	AAAFP	17.480	475.243	476.253	7.990		Non-umami	0.303	0.043
270	LGSV		374.217	375.224	5.610	90	Non-umami	0.302	0.443
271	LMLP		472.272	473.278	10.460	94	Non-umami	0.3	0.01
272	VGSAL	19.110	445.254	446.261	6.730		Non-umami	0.298	0.966
273	AFTPIQ	20.330	675.359	676.369	8.710		Non-umami	0.296	0.993
274	TVGLI	16.800	501.316	502.324	10.950		Non-umami	0.293	0.06
275	PNGDLH	18.220	651.298	652.307	2.750		Non-umami	0.293	0.983
276	LQPQNP	15.230	695.360	696.371	5.180		Non-umami	0.289	0.973
277	VIASI	18.280	501.316	502.326	8.750		Non-umami	0.288	0.106
278	LQPQP		581.317	582.327	5.720	95	Non-umami	0.286	0.477
279	TPIQP	15.560	554.306	555.315	6.800		Non-umami	0.284	0.008
280	TADLPSKKG	20.580	915.503	458.761	2.720		Non-umami	0.282	0.976
281	IALSI	15.390	515.332	516.341	11.090		Non-umami	0.281	0.096
282	VAVRATP	16.360	712.423	713.433	5.290		Non-umami	0.279	0.985
283	LPSIQ	15.170	556.322	557.331	8.160		Non-umami	0.278	0.949
284	VAGSI	16.600	445.254	446.261	6.640		Non-umami	0.272	0.701
285	TVGGI	16.090	445.254	446.262	5.770		Non-umami	0.272	0.207
286	IIQAA	15.480	514.312	515.320	6.450		Non-umami	0.272	0.023
287	VALSI	18.620	501.316	502.325	10.340		Non-umami	0.269	0.235
288	PVSQP	15.600	526.275	527.283	4.270		Non-umami	0.269	0.987
289	ALTVA	18.180	473.285	474.295	8.110		Non-umami	0.268	0.831
290	DRLQ		530.281	531.291	2.270	99	Non-umami	0.267	0.982
291	TPTVG	15.080	473.249	474.258	4.970		Non-umami	0.266	0.955
292	PQQFPQQ	17.350	871.419	872.430	5.910		Non-umami	0.265	0.989
293	LGALP	15.170	469.290	470.299	8.870		Non-umami	0.264	0.053
294	QPQYPQ	16.490	759.355	760.367	4.730		Non-umami	0.261	0.962
295	PLVNP		538.312	539.321	7.750	98	Non-umami	0.259	0.529
296	YPRTP		632.328	317.172	4.340	95	Non-umami	0.256	0.981
297	SIVGL	20.410	487.301	488.310	9.070		Non-umami	0.256	0.011
298	LPHT		466.254	467.262	5.530	94	Non-umami	0.256	0.94
299	LGVD		402.211	403.220	5.930	92	Non-umami	0.256	0.982
300	GISSI	18.870	475.264	476.274	7.880		Non-umami	0.255	0.989
301	VGGPSVG		571.297	572.306	6.190	96	Non-umami	0.25	0.989
302	VELIN	15.200	586.333	587.342	8.880		Non-umami	0.246	0.98
303	FVAGL	15.070	505.290	506.298	9.890		Non-umami	0.246	0.015
304	AILSI	19.520	515.332	516.341	11.090		Non-umami	0.246	0.05
305	ADRHGEGGVA	16.620	1009.458	505.737	3.460		Non-umami	0.246	0.977
306	KGGVD	15.570	474.244	475.251	1.780		Non-umami	0.245	0.99
307	VTALRTIP	21.020	869.533	870.544	9.240		Non-umami	0.244	0.979
308	VVVSPP		596.353	597.364	7.970	97	Non-umami	0.242	0.07
309	ISGAL	18.080	459.269	460.279	9.310		Non-umami	0.241	0.066
310	TAALI	18.200	487.301	488.309	6.100		Non-umami	0.24	0.068
311	TAGLP	18.740	457.254	458.261	6.710		Non-umami	0.239	0.185
312	IAHGGVIPNIN	30.620	1103.609	552.814	8.600		Non-umami	0.238	0.458
313	VTVGL	20.000	487.301	488.310	8.990		Non-umami	0.237	0.967
314	IPSLQ	15.170	556.322	557.331	8.160		Non-umami	0.237	0.95
315	HGAQIP	15.610	621.323	622.333	4.350		Non-umami	0.236	0.156
316	LLQAA	15.980	514.312	515.320	6.450		Non-umami	0.233	0.015
317	FQPQQP	17.680	743.360	744.370	6.200		Non-umami	0.233	0.991
318	ALGAI	17.130	443.274	444.283	7.600		Non-umami	0.224	0.027
319	TVAAL	15.960	473.285	474.293	7.550		Non-umami	0.222	0.831
320	LPLGAP		566.343	567.352	8.990	94	Non-umami	0.222	0.052
321	LPTH		466.254	467.262	5.620	98	Non-umami	0.219	0.743
322	YTNP		493.217	494.225	3.990	98	Non-umami	0.218	0.98
323	TLGAP		457.254	458.263	5.900	92	Non-umami	0.217	0.138
324	LPAGV	17.920	455.274	456.283	7.550	97	Non-umami	0.212	0.048
325	KFTSS	18.390	568.286	569.294	2.360	96	Non-umami	0.212	0.986
326	VVTGVG	16.000	530.306	531.316	5.900		Non-umami	0.211	0.972
327	KVLP		455.311	456.319	5.660	96	Non-umami	0.211	0.012
328	EALR		487.275	488.285	2.360	98	Non-umami	0.211	0.939
329	VPVE		442.243	443.252	5.110	92	Non-umami	0.208	0.936
330	TLLL		458.310	459.320	11.280	92	Non-umami	0.208	0.012
331	TLGGLP	15.270	556.322	557.332	8.600		Non-umami	0.208	0.164
332	GSILL	18.680	501.316	502.325	11.050		Non-umami	0.208	0.031
333	GLVGE	15.370	473.249	474.258	6.100		Non-umami	0.208	0.981
334	LSIAI	16.100	515.332	516.341	10.360		Non-umami	0.204	0.041
335	PQQIPPQ	20.400	806.429	807.439	6.260		Non-umami	0.202	0.059
336	FDLR		549.291	550.302	8.340	94	Non-umami	0.201	0.993
337	PQQVPPQ	22.680	792.413	793.420	5.560		Non-umami	0.2	0.947
338	LGGISS	17.300	532.286	533.295	6.450		Non-umami	0.2	0.987
339	LLVAP	17.980	511.337	512.346	9.830		Non-umami	0.199	0.008
340	LEPL		470.274	471.283	8.950	92	Non-umami	0.198	0.286
341	ALTGL	16.280	473.285	474.294	7.640		Non-umami	0.198	0.965
342	MPLEGQ		673.311	674.320	6.840	94	Non-umami	0.197	0.977
343	LVATP	15.240	499.301	500.309	6.580		Non-umami	0.196	0.392
344	VLASL	18.280	501.316	502.326	8.750		Non-umami	0.195	0.476
345	WMPLE		716.320	717.334	10.150	99	Non-umami	0.193	0.097
346	VMLP		458.256	459.265	9.640	90	Non-umami	0.192	0.01
347	LTAVFP		646.369	647.379	11.540	91	Non-umami	0.192	0.111
348	GVSAL	17.270	445.254	446.261	6.730		Non-umami	0.192	0.966
349	VAGIP	16.130	455.274	456.283	8.330		Non-umami	0.191	0.047
350	VAAGVLP	15.060	625.380	626.390	9.230		Non-umami	0.191	0.184
351	FVAGI	15.030	505.290	506.298	9.890		Non-umami	0.189	0.014
352	TLFPLNL		816.475	817.485	14.360	90	Non-umami	0.188	0.984
353	TAAIL	18.200	487.301	488.309	6.100		Non-umami	0.188	0.038
354	LVAIP	16.500	511.337	512.347	11.790		Non-umami	0.188	0.042
355	VAISL	18.620	501.316	502.325	10.340		Non-umami	0.187	0.261
356	LAGAL	20.000	443.274	444.283	7.600		Non-umami	0.186	0.116
357	LALAL	16.900	499.337	500.346	11.530	95	Non-umami	0.185	0.059
358	LGAPL	15.160	469.290	470.299	8.220		Non-umami	0.183	0.024
359	AITVA	18.180	473.285	474.295	8.110		Non-umami	0.183	0.427
360	LTTSR	15.120	576.323	577.334	2.240		Non-umami	0.181	0.98
361	LGVP		384.237	385.246	7.970	95	Non-umami	0.181	0.029
362	VPSQP	17.590	526.275	527.283	4.270	99	Non-umami	0.18	0.967
363	TVLVP	16.140	527.332	528.341	8.590		Non-umami	0.178	0.015
364	TLAGI	18.800	473.285	474.293	7.160		Non-umami	0.178	0.083
365	AVLSL	19.770	501.316	502.325	10.260		Non-umami	0.178	0.546
366	QPYPQQP	22.510	856.408	857.419	6.030		Non-umami	0.177	0.96
367	LSVQ		445.254	446.262	5.500	91	Non-umami	0.174	0.971
368	TVGLL	16.800	501.316	502.324	10.950		Non-umami	0.171	0.221
369	VATLFPLGGL	17.730	986.580	494.298	15.000		Non-umami	0.169	0.974
370	ATGAL	15.910	431.238	432.246	4.650		Non-umami	0.167	0.939
371	LPLQP		566.343	567.352	8.600	93	Non-umami	0.166	0.096
372	LSLE		460.253	461.262	7.120	90	Non-umami	0.165	0.978
373	INDIFEKLA	19.570	1061.576	531.797	10.610		Non-umami	0.165	0.978
374	VPQQRP		723.403	362.710	2.750	94	Non-umami	0.164	0.983
375	FSPVLVP	17.670	757.437	758.447	12.120		Non-umami	0.162	0.179
376	LTVAGP		556.322	557.331	7.610	96	Non-umami	0.158	0.976
377	ISAVF	15.670	535.301	536.310	10.630		Non-umami	0.158	0.102
378	VIASL	18.280	501.316	502.326	8.750		Non-umami	0.157	0.07
379	AIGAL	16.630	443.274	444.283	7.600		Non-umami	0.157	0.039
380	VVPP		410.253	411.262	6.100	96	Non-umami	0.156	0.009
381	QVGAL	15.490	487.264	488.272	7.210		Non-umami	0.156	0.173
382	FPGAS	15.750	477.222	478.233	5.330	93	Non-umami	0.155	0.845
383	HPGQQ	16.420	565.261	566.268	1.890		Non-umami	0.151	0.987
384	FGKEP		576.291	577.300	9.230	93	Non-umami	0.151	0.289
385	HQPGQ		565.261	566.269	1.990	93	Non-umami	0.15	0.991
386	LFSPV	15.750	561.316	562.326	10.070	93	Non-umami	0.149	0.654
387	ALLP		412.269	413.277	9.340	98	Non-umami	0.149	0.008
388	IGGLSS	17.300	532.286	533.295	6.450		Non-umami	0.148	0.986
389	LPEDAKVE	19.470	899.460	450.739	5.960	98	Non-umami	0.147	0.976
390	LLPQQ		597.349	598.357	5.850	96	Non-umami	0.146	0.983
391	FPQQQP	15.450	743.360	744.371	6.130		Non-umami	0.146	0.994
392	AFTPIQY	24.470	838.423	839.433	10.130		Non-umami	0.145	0.847
393	LLPHT		579.338	580.346	7.100	94	Non-umami	0.144	0.73
394	TAAII	15.060	487.301	488.309	6.100		Non-umami	0.143	0.029
395	AGALL	15.040	443.274	444.283	8.510		Non-umami	0.143	0.021
396	TGLP		386.217	387.225	6.560	95	Non-umami	0.141	0.119
397	PTGSMGGE	15.540	734.291	735.300	3.380		Non-umami	0.138	0.978
398	PSGQVQW	17.860	800.382	801.392	8.040		Non-umami	0.138	0.989
399	AGALI	15.040	443.274	444.283	8.510		Non-umami	0.136	0.032
400	TVVGI	15.750	487.301	488.310	8.920		Non-umami	0.135	0.073
401	ISGAI	15.210	459.269	460.279	9.310		Non-umami	0.135	0.106
402	PFLGSGLAGL	16.320	930.518	466.268	12.780		Non-umami	0.134	0.967
403	LALP		412.269	413.277	9.430	98	Non-umami	0.134	0.041
404	KLSL		501.316	502.325	10.260	95	Non-umami	0.134	0.953
405	ILVAG	16.500	471.306	472.315	8.310		Non-umami	0.133	0.025
406	AVATP	16.400	457.254	458.262	5.200		Non-umami	0.133	0.974
407	GSLII	18.680	501.316	502.325	11.050		Non-umami	0.132	0.044
408	SPVLVPAA	19.670	752.443	753.453	9.680		Non-umami	0.131	0.029
409	FSGAP	17.110	477.222	478.232	5.460		Non-umami	0.131	0.362
410	QPQVPP	17.890	664.354	665.362	5.610		Non-umami	0.129	0.053
411	AIISL	19.520	515.332	516.341	11.090		Non-umami	0.129	0.066
412	MPMP		474.197	475.206	8.310	97	Non-umami	0.128	0.008
413	EIQTSVR	15.830	831.445	416.731	5.010		Non-umami	0.127	0.975
414	ISLAI	16.100	515.332	516.341	10.360		Non-umami	0.125	0.034
415	ELGGI	15.930	487.264	488.273	7.060		Non-umami	0.125	0.872
416	TVAAI	15.960	473.285	474.293	7.550		Non-umami	0.124	0.099
417	TFPQQP	17.790	716.349	717.356	7.140		Non-umami	0.124	0.983
418	GAHVTMH	17.880	751.344	752.348	5.230		Non-umami	0.124	0.986
419	ALISI	19.520	515.332	516.341	11.090		Non-umami	0.124	0.051
420	TLFPL	15.270	589.348	590.356	13.090		Non-umami	0.123	0.105
421	KATPVF		703.390	704.400	10.250	91	Non-umami	0.123	0.948
422	PMAP		430.189	431.197	3.370	98	Non-umami	0.121	0.042
423	AAGGIGQP	17.120	669.345	670.355	5.920		Non-umami	0.121	0.921
424	ALTGI	16.280	473.285	474.294	7.640		Non-umami	0.12	0.684
425	AAEGSIL	16.830	659.349	660.359	8.500		Non-umami	0.12	0.978
426	YVVLP	16.420	589.348	590.357	10.520		Non-umami	0.119	0.012
427	QPFQQP	16.280	743.360	744.367	6.650		Non-umami	0.117	0.996
428	LLPFT		589.348	590.357	11.600	90	Non-umami	0.117	0.242
429	IAGAL	20.000	443.274	444.283	7.600		Non-umami	0.117	0.033
430	LVQVP		554.343	555.352	9.340	91	Non-umami	0.116	0.049
431	LATGL	18.190	473.285	474.294	8.200		Non-umami	0.116	0.961
432	FGGSP	18.970	463.207	464.216	5.310		Non-umami	0.116	0.384
433	VVGL		386.253	387.261	8.220	98	Non-umami	0.115	0.008
434	VAGVP	16.180	441.259	442.267	7.080	97	Non-umami	0.115	0.447
435	TGALAI	15.780	544.322	545.332	7.960		Non-umami	0.115	0.767
436	LVVPAAL		681.443	682.453	11.570	91	Non-umami	0.115	0.019
437	LAGALE	15.120	572.317	573.325	6.670		Non-umami	0.115	0.983
438	ISIAL	16.100	515.332	516.341	10.360		Non-umami	0.115	0.039
439	VAGSL	18.800	445.254	446.261	6.640		Non-umami	0.114	0.957
440	LTLGM		549.283	550.291	7.300	97	Non-umami	0.114	0.854
441	LSGAV	19.220	445.254	446.262	6.540	93	Non-umami	0.114	0.43
442	YPQQP	16.800	631.297	632.306	5.440		Non-umami	0.113	0.982
443	TVGGL	16.090	445.254	446.262	5.770		Non-umami	0.113	0.681
444	TPVSF	16.370	549.280	550.290	8.730		Non-umami	0.112	0.991
445	LLPTH		579.338	580.346	7.200	96	Non-umami	0.112	0.784
446	GMGLPSNP	17.010	771.359	772.368	8.350		Non-umami	0.111	0.981
447	LSGV		374.217	375.225	5.870	94	Non-umami	0.109	0.101
448	YPQNP	18.710	617.281	618.291	4.860		Non-umami	0.108	0.98
449	TVIGA	15.410	459.269	460.278	6.970		Non-umami	0.108	0.062
450	SAVVGL	15.520	544.322	545.332	9.070		Non-umami	0.108	0.574
451	LTGL		402.248	403.257	7.900	98	Non-umami	0.108	0.884
452	AVISL	19.770	501.316	502.325	10.260		Non-umami	0.108	0.099
453	TIVVAP	18.130	598.369	599.379	8.710		Non-umami	0.107	0.046
454	IVVAP	15.100	497.321	498.331	8.550		Non-umami	0.107	0.011
455	TLAGP	16.830	457.254	458.263	5.810	93	Non-umami	0.106	0.238
456	QSHP		467.213	468.222	1.960	91	Non-umami	0.106	0.96
457	PVFSF		595.301	596.310	11.600	91	Non-umami	0.106	0.164
458	GSILI	15.460	501.316	502.325	11.050		Non-umami	0.104	0.041
459	FQAGP		518.249	519.257	5.570	97	Non-umami	0.104	0.037
460	TLTSR	16.560	576.323	577.334	2.240		Non-umami	0.103	0.979
461	SGAPVYL	21.690	705.370	706.380	9.680		Non-umami	0.103	0.456
462	VLFSP	17.510	561.316	562.326	9.790		Non-umami	0.102	0.249
463	PTVSF		549.280	550.290	8.730	99	Non-umami	0.102	0.989
464	DLSK		461.249	462.256	2.120	94	Non-umami	0.102	0.982
465	QPFPQQ	16.230	743.360	744.370	6.430		Non-umami	0.1	0.993
466	LSGLL	17.220	501.316	502.324	10.950		Non-umami	0.099	0.138
467	IPQQP	18.010	581.317	582.325	5.570		Non-umami	0.099	0.681
468	LVSGAVIP	16.070	754.459	755.469	10.070		Non-umami	0.098	0.961
469	LIVGV	16.290	499.337	500.345	10.820		Non-umami	0.098	0.01
470	LGGDGVFKQLQR		1316.720	439.916	7.890	90	Non-umami	0.098	0.984
471	AVISI	17.620	501.316	502.325	10.260		Non-umami	0.098	0.212
472	QPYPQ	15.990	631.297	632.307	5.130		Non-umami	0.097	0.935
473	LLGAN	15.430	486.280	487.288	6.650	90	Non-umami	0.097	0.976
474	ITGAP	15.400	457.254	458.263	5.980		Non-umami	0.097	0.013
475	AGAIL	15.040	443.274	444.283	8.510		Non-umami	0.097	0.038
476	LALAI	16.900	499.337	500.346	11.530		Non-umami	0.096	0.022
477	IAHGGVIP	15.340	762.439	382.228	7.380		Non-umami	0.096	0.065
478	LLVGV	16.290	499.337	500.345	10.820		Non-umami	0.095	0.007
479	LAIAL	16.900	499.337	500.346	11.530		Non-umami	0.094	0.033
480	AVLSI	19.770	501.316	502.325	10.260		Non-umami	0.094	0.283
481	VLASI	18.280	501.316	502.326	8.750		Non-umami	0.093	0.287
482	VLVPAAL	16.990	681.443	682.453	11.570		Non-umami	0.092	0.015
483	TVAGAL	15.670	530.306	531.315	7.410		Non-umami	0.092	0.974
484	LLPQQP		694.401	695.410	7.170	96	Non-umami	0.092	0.882
485	VIVAP	15.120	497.321	498.330	8.280		Non-umami	0.091	0.011
486	PFLGSGLAGLL	15.330	1043.601	522.810	14.830		Non-umami	0.091	0.93
487	LLVAG	16.500	471.306	472.315	8.310		Non-umami	0.091	0.018
488	ALGLP	16.890	469.290	470.299	9.730		Non-umami	0.091	0.022
489	VGVVF	15.490	519.306	520.315	10.760		Non-umami	0.09	0.017
490	SVGV		360.201	361.209	5.490	98	Non-umami	0.09	0.447
491	IFSPV	15.460	561.316	562.326	10.070		Non-umami	0.089	0.205
492	ERFQPMF	17.520	953.443	477.730	9.620		Non-umami	0.089	0.851
493	AIGGLTQL	15.990	771.449	772.459	10.760		Non-umami	0.089	0.974
494	PLLQP		566.343	567.353	8.690	96	Non-umami	0.088	0.048
495	ELGGL	15.930	487.264	488.273	7.060		Non-umami	0.088	0.954
496	PSGQVQWP	15.120	897.434	898.446	9.240		Non-umami	0.087	0.65
497	AAGGL	17.380	387.212	388.219	4.550		Non-umami	0.087	0.071
498	TLFPLGGL	18.140	816.475	817.485	14.360		Non-umami	0.086	0.352
499	TLAAGP		528.291	529.301	6.180	92	Non-umami	0.086	0.4
500	LQNGP		527.270	528.280	5.780	91	Non-umami	0.086	0.981
501	VLPPVEP	15.130	749.432	750.442	9.520		Non-umami	0.085	0.018
502	IEAVP	15.980	527.296	528.305	8.600		Non-umami	0.085	0.932
503	FPQQ		518.249	519.257	5.200	98	Non-umami	0.085	0.704
504	VVLP		426.284	427.293	9.160	96	Non-umami	0.084	0.01
505	LPQQPP	23.030	678.370	679.381	6.390		Non-umami	0.084	0.376
506	LPPQQP	17.540	678.370	679.381	6.080		Non-umami	0.084	0.766
507	LPAGL	15.730	469.290	470.299	9.170		Non-umami	0.084	0.039
508	ASVVGL	15.910	544.322	545.331	9.170		Non-umami	0.084	0.973
509	VLAVP		497.321	498.331	9.200	97	Non-umami	0.083	0.028
510	TVAGI	15.430	459.269	460.278	7.740		Non-umami	0.082	0.351
511	LTGAP	15.400	457.254	458.263	5.980		Non-umami	0.082	0.254
512	LLLKVN		698.469	350.243	8.540	91	Non-umami	0.082	0.971
513	LIVAP	17.160	511.337	512.346	9.830		Non-umami	0.082	0.01
514	LAGAP	16.830	427.243	428.251	5.660	98	Non-umami	0.082	0.12
515	VLEGK		544.322	545.331	2.610	92	Non-umami	0.081	0.977
516	ISGAV	19.220	445.254	446.262	6.540		Non-umami	0.081	0.061
517	YPQP		503.238	504.247	5.920	93	Non-umami	0.078	0.038
518	TSPHQP	16.350	665.313	666.323	2.190		Non-umami	0.078	0.968
519	TLGGIP	15.460	556.322	557.332	8.600		Non-umami	0.078	0.031
520	QQPIP	17.730	581.317	582.327	7.000		Non-umami	0.078	0.034
521	GVAFP	16.120	489.259	490.267	9.540		Non-umami	0.078	0.023
522	FPFP		506.253	507.262	11.870	95	Non-umami	0.078	0.019
523	VEIIN	15.200	586.333	587.342	8.880		Non-umami	0.077	0.979
524	TLPTM		577.278	578.287	5.760	93	Non-umami	0.077	0.883
525	LGPF		432.237	433.246	9.830	94	Non-umami	0.077	0.019
526	GVSAI	17.270	445.254	446.261	6.730		Non-umami	0.077	0.593
527	VLVPAAI	16.990	681.443	682.453	11.570		Non-umami	0.076	0.018
528	LQYVHP		755.397	378.706	6.600	96	Non-umami	0.076	0.746
529	ILGAN	15.430	486.280	487.288	6.650		Non-umami	0.076	0.964
530	FGPF		466.222	467.231	10.550	97	Non-umami	0.076	0.02
531	AITGI	16.280	473.285	474.294	7.640		Non-umami	0.076	0.298
532	VAAK		387.248	388.256	1.930	97	Non-umami	0.075	0.028
533	SKYNN	16.700	624.287	625.292	1.780		Non-umami	0.075	0.982
534	VGGPSVGV		670.365	671.375	8.140	95	Non-umami	0.074	0.99
535	FVVVP	15.970	559.337	560.345	10.970		Non-umami	0.074	0.019
536	AITGL	16.280	473.285	474.294	7.640		Non-umami	0.074	0.449
537	ALLGF	16.650	519.306	520.315	11.760		Non-umami	0.073	0.014
538	LGPFL		545.321	546.330	12.130	96	Non-umami	0.072	0.017
539	TIAAGL	16.550	544.322	545.332	8.580		Non-umami	0.071	0.871
540	LPFP		472.269	473.277	10.890	97	Non-umami	0.071	0.019
541	AGPK		371.217	372.224	1.670	95	Non-umami	0.071	0.03
542	AAAIT	19.980	445.254	446.262	5.960		Non-umami	0.071	0.973
543	AAAAFP	19.760	546.280	547.290	8.320	90	Non-umami	0.071	0.061
544	TGAFP	15.080	491.238	492.247	7.330		Non-umami	0.07	0.128
545	IVKVTP	16.010	655.427	656.436	6.890		Non-umami	0.07	0.941
546	VLLGP	15.170	497.321	498.331	9.110	92	Non-umami	0.069	0.008
547	LVLSGL	17.170	600.385	601.394	11.240		Non-umami	0.069	0.885
548	ISNLQ	16.370	573.312	574.322	6.550		Non-umami	0.069	0.982
549	VLLSGL	16.230	600.385	601.394	11.330	96	Non-umami	0.068	0.855
550	AHGPGQW	15.320	751.340	752.350	5.140		Non-umami	0.068	0.985
551	LLFP		488.300	489.309	11.810	95	Non-umami	0.067	0.017
552	FPGGL		489.259	490.268	9.300	95	Non-umami	0.067	0.02
553	FGFP		466.222	467.232	10.650	94	Non-umami	0.067	0.02
554	VIFSP	17.510	561.316	562.326	9.790		Non-umami	0.066	0.18
555	LGFP		432.237	433.245	9.930	96	Non-umami	0.066	0.021
556	GAAGL	15.170	387.212	388.219	4.550		Non-umami	0.066	0.069
557	ALGL		372.237	373.246	8.930	92	Non-umami	0.066	0.042
558	NLALQTL	15.020	771.449	772.459	10.680		Non-umami	0.065	0.976
559	LPGVL	18.790	497.321	498.330	10.340	99	Non-umami	0.065	0.008
560	LGGL		358.222	359.230	7.650	98	Non-umami	0.065	0.055
561	LAGAI	20.000	443.274	444.283	7.600		Non-umami	0.065	0.039
562	TIAGL	18.280	473.285	474.293	7.160		Non-umami	0.064	0.087
563	IALAL	16.900	499.337	500.346	11.530		Non-umami	0.064	0.02
564	FLPFP		619.337	620.346	13.340	98	Non-umami	0.064	0.019
565	ALLL		428.300	429.308	10.570	91	Non-umami	0.064	0.006
566	PQQLPP	19.780	678.370	679.380	6.480		Non-umami	0.063	0.588
567	INDIFEKL	15.200	990.539	496.277	10.610		Non-umami	0.063	0.978
568	IGALP	15.340	469.290	470.299	8.870		Non-umami	0.063	0.04
569	AAEGSII	20.380	659.349	660.359	8.500		Non-umami	0.063	0.978
570	VSVSH	15.960	527.270	528.279	2.420		Non-umami	0.062	0.99
571	TIAGI	18.800	473.285	474.293	7.160		Non-umami	0.062	0.047
572	ILPQQP	17.010	694.401	695.410	7.170		Non-umami	0.062	0.856
573	VLSGL	18.730	487.301	488.310	8.730		Non-umami	0.061	0.575
574	LVVAP	15.100	497.321	498.331	8.550	92	Non-umami	0.061	0.011
575	LLGGL	19.970	471.306	472.314	10.180	95	Non-umami	0.061	0.134
576	LGGLL	17.670	471.306	472.315	10.440		Non-umami	0.061	0.035
577	IVAIP	16.500	511.337	512.347	11.790		Non-umami	0.061	0.045
578	FPPQ		487.243	488.253	6.430	96	Non-umami	0.061	0.01
579	FPPP		456.237	457.249	7.460	94	Non-umami	0.061	0.019
580	WMLP		587.278	588.289	10.970	99	Non-umami	0.06	0.018
581	SGAPVY	19.600	592.286	593.295	6.400		Non-umami	0.06	0.738
582	PVGPTPP	22.090	663.359	664.368	7.390		Non-umami	0.06	0.272
583	PVGPPTP	23.430	663.359	664.368	7.390		Non-umami	0.06	0.274
584	VQPP		439.243	440.252	5.370	93	Non-umami	0.059	0.061
585	SPAGAGFP	15.330	702.334	703.343	6.450		Non-umami	0.059	0.184
586	LLNP		455.274	456.283	7.220	98	Non-umami	0.059	0.081
587	KLAP		427.279	428.288	3.890	93	Non-umami	0.059	0.015
588	IPGGL	19.010	455.274	456.284	8.830		Non-umami	0.059	0.012
589	PFRPP		612.338	613.348	6.930	98	Non-umami	0.058	0.02
590	LNDLFEKI	15.200	990.539	496.277	10.610		Non-umami	0.058	0.978
591	LLLAG	16.550	485.321	486.330	9.270		Non-umami	0.058	0.015
592	LFALP	15.090	559.337	560.346	11.880		Non-umami	0.058	0.014
593	LALGF		519.306	520.315	11.760	94	Non-umami	0.058	0.048
594	IAGAP	17.570	427.243	428.251	5.660		Non-umami	0.058	0.045
595	LVGGL	19.340	457.290	458.299	8.920	91	Non-umami	0.057	0.06
596	LLGF		448.269	449.278	11.300	99	Non-umami	0.057	0.016
597	PIGGL	17.310	455.274	456.284	8.580		Non-umami	0.056	0.017
598	TIFPI	15.270	589.348	590.356	13.090		Non-umami	0.055	0.14
599	LIVAG	16.500	471.306	472.315	8.310		Non-umami	0.055	0.033
600	LGLGL	18.240	471.306	472.315	11.800		Non-umami	0.055	0.173
601	LAGL		372.237	373.245	7.240	94	Non-umami	0.055	0.049
602	GLLAL	15.360	485.321	486.331	10.400		Non-umami	0.055	0.017
603	GLALL	15.250	485.321	486.330	10.210		Non-umami	0.055	0.017
604	AIISI	19.520	515.332	516.341	11.090		Non-umami	0.055	0.137
605	AAANVP	15.510	541.286	542.296	6.940		Non-umami	0.055	0.982
606	AAALT	19.980	445.254	446.262	5.960		Non-umami	0.055	0.981
607	VLVAP	15.120	497.321	498.330	8.280		Non-umami	0.054	0.011
608	TIFPL	15.270	589.348	590.356	13.090		Non-umami	0.054	0.133
609	LVGGI	19.340	457.290	458.299	8.920		Non-umami	0.054	0.026
610	LLWP		527.311	528.321	11.770	93	Non-umami	0.054	0.017
611	LGLL		414.284	415.292	10.800	97	Non-umami	0.054	0.008
612	FRLP		531.317	532.326	8.720	98	Non-umami	0.054	0.018
613	LLIPP	16.900	551.368	552.378	10.500		Non-umami	0.053	0.01
614	IPGGI	19.010	455.274	456.284	8.830		Non-umami	0.053	0.013
615	FPWQ		576.270	577.279	10.090	99	Non-umami	0.053	0.014
616	FPSQQP	16.800	702.334	703.344	6.220		Non-umami	0.053	0.988
617	FPRPP		612.338	307.177	6.980	98	Non-umami	0.053	0.02
618	VLSGI	18.730	487.301	488.310	8.730		Non-umami	0.052	0.56
619	VILGP	15.170	497.321	498.331	9.110		Non-umami	0.052	0.01
620	LIIPP	16.900	551.368	552.378	10.500		Non-umami	0.052	0.012
621	LFPL		488.300	489.309	12.310	93	Non-umami	0.052	0.019
622	IATGL	18.190	473.285	474.294	8.200		Non-umami	0.052	0.409
623	AFEPLSR		818.429	410.223	7.020	92	Non-umami	0.052	0.973
624	VLPP		424.269	425.277	6.970	96	Non-umami	0.051	0.009
625	LELGS	16.380	517.275	518.283	6.750		Non-umami	0.051	0.979
626	IIIPP	16.900	551.368	552.378	10.500		Non-umami	0.051	0.012
627	IATGI	18.190	473.285	474.294	8.200		Non-umami	0.051	0.413
628	ALAASVVG	15.970	686.396	687.407	8.110		Non-umami	0.051	0.982
629	VLLPP	15.080	537.353	538.362	9.790	91	Non-umami	0.05	0.008
630	VILSGI	16.230	600.385	601.394	11.330		Non-umami	0.05	0.858
631	LLLPP	16.900	551.368	552.378	10.500		Non-umami	0.05	0.008
632	FLLIP	18.130	601.384	602.392	13.950		Non-umami	0.05	0.017
633	VLLP		440.300	441.308	10.640	98	Non-umami	0.049	0.009
634	PFTQPQ	15.280	716.349	717.360	7.030		Non-umami	0.049	0.987
635	LVLGP	16.410	497.321	498.331	9.110		Non-umami	0.049	0.008
636	LSGLI	17.220	501.316	502.324	10.950		Non-umami	0.049	0.043
637	FPSQP	17.360	574.275	575.285	6.850	96	Non-umami	0.049	0.99
638	FGPTGL	15.780	590.306	591.316	9.550	99	Non-umami	0.049	0.72
639	VVPFQ	18.720	630.338	631.347	12.560		Non-umami	0.048	0.042
640	PIGGI	19.700	455.274	456.284	8.580		Non-umami	0.048	0.018
641	LATGI	18.190	473.285	474.294	8.200		Non-umami	0.048	0.647
642	IPGVL	15.270	497.321	498.330	10.340		Non-umami	0.048	0.008
643	IPGVI	15.270	497.321	498.330	10.340		Non-umami	0.048	0.01
644	IGGLL	17.670	471.306	472.315	10.440		Non-umami	0.048	0.013
645	IGGLI	17.670	471.306	472.315	10.440		Non-umami	0.048	0.011
646	VPILQ	15.110	568.358	569.368	9.390		Non-umami	0.047	0.056
647	VFPL		474.284	475.294	11.460	95	Non-umami	0.047	0.019
648	QIIPQQP	15.110	822.460	823.470	8.180		Non-umami	0.047	0.483
649	LLAGP	15.960	469.290	470.299	7.540	95	Non-umami	0.047	0.026
650	ISGLL	17.220	501.316	502.324	10.950		Non-umami	0.047	0.038
651	IIGGL	19.970	471.306	472.314	10.180		Non-umami	0.047	0.011
652	GPALF	17.130	503.274	504.282	9.910		Non-umami	0.047	0.05
653	FPWQP	16.130	673.322	674.331	10.810	98	Non-umami	0.047	0.014
654	FLAGP	15.110	503.274	504.283	8.330		Non-umami	0.047	0.057
655	VVTGVGGQ		715.386	716.396	5.680	97	Non-umami	0.046	0.968
656	LVVAAP		568.358	569.368	8.750	91	Non-umami	0.046	0.043
657	LPYVHP		724.391	363.204	7.840	97	Non-umami	0.046	0.01
658	YPSQ		493.217	494.225	4.080	98	Non-umami	0.045	0.982
659	LGGLI	17.670	471.306	472.315	10.440		Non-umami	0.045	0.012
660	LFLIP	18.290	601.384	602.392	13.950		Non-umami	0.045	0.017
661	KAPP		411.248	412.257	2.100	98	Non-umami	0.045	0.014
662	ILGGL	19.970	471.306	472.314	10.180		Non-umami	0.045	0.011
663	IAGAI	20.000	443.274	444.283	7.600		Non-umami	0.045	0.044
664	FPLQ		503.274	504.284	8.820	98	Non-umami	0.045	0.018
665	FLLLP	18.130	601.384	602.392	13.950		Non-umami	0.045	0.017
666	AGAII	15.040	443.274	444.283	8.510		Non-umami	0.045	0.047
667	LGGAV	15.990	415.243	416.251	6.610	91	Non-umami	0.044	0.06
668	GPLW		471.248	472.258	9.750	96	Non-umami	0.044	0.016
669	FPQP		487.243	488.252	7.500	95	Non-umami	0.044	0.014
670	FGLP		432.237	433.246	10.180	96	Non-umami	0.044	0.02
671	APPPP	23.460	477.259	478.268	4.810	99	Non-umami	0.044	0.012
672	VFLP		474.284	475.293	11.390	97	Non-umami	0.043	0.018
673	LPGVI	17.370	497.321	498.330	10.340		Non-umami	0.043	0.009
674	LPGGL	17.160	455.274	456.284	8.830	98	Non-umami	0.043	0.044
675	LLGGI	19.970	471.306	472.314	10.180		Non-umami	0.043	0.012
676	IIVAG	16.500	471.306	472.315	8.310		Non-umami	0.043	0.039
677	TLGGL	19.510	459.269	460.278	8.680		Non-umami	0.042	0.858
678	LIGGL	19.970	471.306	472.314	10.180		Non-umami	0.042	0.013
679	LAAGP	15.080	427.243	428.251	5.260		Non-umami	0.042	0.12
680	IPGLP	15.500	495.306	496.315	10.170		Non-umami	0.042	0.01
681	IAIAL	16.900	499.337	500.346	11.530		Non-umami	0.042	0.034
682	SLGGL	20.400	445.254	446.262	7.030		Non-umami	0.041	0.154
683	LSNLQ	16.370	573.312	574.322	6.550		Non-umami	0.041	0.982
684	LPGGI	19.010	455.274	456.284	8.830		Non-umami	0.041	0.012
685	LLQP		469.290	470.298	6.910	90	Non-umami	0.041	0.051
686	LLLP		454.316	455.324	11.270	97	Non-umami	0.041	0.008
687	KIQPFP	16.590	728.422	729.432	8.370		Non-umami	0.041	0.012
688	IGGAV	16.360	415.243	416.251	6.610		Non-umami	0.041	0.039
689	FLIIP	18.130	601.384	602.392	13.950		Non-umami	0.041	0.017
690	FALAGP	15.100	574.312	575.322	9.300		Non-umami	0.041	0.054
691	SYPQPP	20.280	687.323	688.332	6.930		Non-umami	0.04	0.023
692	PLGGL	19.700	455.274	456.284	8.580	99	Non-umami	0.04	0.078
693	LQGGGP		527.270	528.279	5.670	92	Non-umami	0.04	0.714
694	LPAGLP	15.410	566.343	567.352	9.750	96	Non-umami	0.04	0.079
695	LGLP		398.253	399.261	8.260	94	Non-umami	0.04	0.016
696	ILLPP	16.960	551.368	552.378	10.500		Non-umami	0.04	0.009
697	ILGGI	18.120	471.306	472.314	10.180		Non-umami	0.04	0.011
698	FPLQPP		697.380	698.389	10.590	90	Non-umami	0.04	0.013
699	FPAGP		487.243	488.253	7.730	98	Non-umami	0.04	0.024
700	AAGGI	17.380	387.212	388.219	4.550		Non-umami	0.04	0.053
701	WQWN		632.271	633.281	9.070	90	Non-umami	0.039	0.038
702	LSGGP	15.300	429.222	430.230	4.520		Non-umami	0.039	0.085
703	LPPEPP		648.348	649.358	7.350	99	Non-umami	0.039	0.04
704	LPAGI	15.730	469.290	470.299	9.170		Non-umami	0.039	0.032
705	LLPQAGP		694.401	695.410	7.320	97	Non-umami	0.039	0.072
706	LIAGP	15.960	469.290	470.299	7.540		Non-umami	0.039	0.042
707	LFLLP	18.290	601.384	602.392	13.950		Non-umami	0.039	0.017
708	KLLP		511.337	512.346	10.780	90	Non-umami	0.039	0.01
709	FIIIP	18.130	601.384	602.392	13.950		Non-umami	0.039	0.019
710	TVGAGL	16.500	516.291	517.299	7.340		Non-umami	0.038	0.946
711	PPPVDH		660.323	331.170	3.460	97	Non-umami	0.038	0.981
712	PAAPFP	17.400	598.312	599.321	8.170	96	Non-umami	0.038	0.014
713	LAGSPVS		629.338	630.348	6.260	94	Non-umami	0.038	0.985
714	IGLGI	18.240	471.306	472.315	11.800		Non-umami	0.038	0.01
715	GLLAI	15.360	485.321	486.331	10.400		Non-umami	0.038	0.022
716	TLGGI	19.510	459.269	460.278	8.680		Non-umami	0.037	0.136
717	LPPP		422.253	423.262	6.240	99	Non-umami	0.037	0.01
718	LGTGP	15.460	443.238	444.248	4.960		Non-umami	0.037	0.82
719	LGLGI	18.240	471.306	472.315	11.800		Non-umami	0.037	0.012
720	LGAIP	15.340	469.290	470.299	8.870		Non-umami	0.037	0.044
721	IPAGV	17.920	455.274	456.283	7.550		Non-umami	0.037	0.034
722	IGIGL	18.240	471.306	472.315	11.800		Non-umami	0.037	0.011
723	ALGIP	16.890	469.290	470.299	9.730		Non-umami	0.037	0.01
724	AIGAI	17.130	443.274	444.283	7.600		Non-umami	0.037	0.049
725	LVLLP	15.050	553.384	554.394	12.630	97	Non-umami	0.036	0.009
726	LIGGI	19.970	471.306	472.314	10.180		Non-umami	0.036	0.012
727	LGGGL	20.350	415.243	416.251	6.950	98	Non-umami	0.036	0.116
728	IPLLP	16.050	551.368	552.378	11.970		Non-umami	0.036	0.009
729	IIGGI	19.970	471.306	472.314	10.180		Non-umami	0.036	0.012
730	IGGII	15.860	471.306	472.315	10.440		Non-umami	0.036	0.012
731	IALAI	18.520	499.337	500.346	11.530		Non-umami	0.036	0.023
732	FPLQP		600.327	601.336	9.880	99	Non-umami	0.036	0.015
733	FIPQIP	18.960	713.411	714.421	11.930		Non-umami	0.036	0.015
734	LPGQ		413.227	414.236	4.060	97	Non-umami	0.035	0.732
735	LLVPV	15.090	539.368	540.377	12.800		Non-umami	0.035	0.007
736	LIGAN	15.430	486.280	487.288	6.650		Non-umami	0.035	0.968
737	LGAGL	16.620	429.259	430.267	8.560		Non-umami	0.035	0.087
738	ISGLI	17.220	501.316	502.324	10.950		Non-umami	0.035	0.054
739	IPAGL	15.730	469.290	470.299	9.170		Non-umami	0.035	0.032
740	IGGIL	17.670	471.306	472.315	10.440		Non-umami	0.035	0.012
741	IFLLP	18.290	601.384	602.392	13.950		Non-umami	0.035	0.017
742	GPPYIA	22.560	616.322	617.333	8.560		Non-umami	0.035	0.008
743	FPPQLP		697.380	698.389	10.910	95	Non-umami	0.035	0.012
744	AVLGL	17.090	471.306	472.315	11.800		Non-umami	0.035	0.009
745	PAPPP	18.310	477.259	478.267	4.620		Non-umami	0.034	0.015
746	LLGGGM		546.284	547.292	8.150	99	Non-umami	0.034	0.081
747	LGAP		356.206	357.213	4.440	98	Non-umami	0.034	0.047
748	LFSGF		569.285	570.295	10.420	93	Non-umami	0.034	0.159
749	LFAIP	15.090	559.337	560.346	11.880		Non-umami	0.034	0.015
750	IPAGLP	15.410	566.343	567.352	9.750		Non-umami	0.034	0.043
751	IGAPI	15.160	469.290	470.299	8.220		Non-umami	0.034	0.01
752	EIGGL	15.930	487.264	488.273	7.060		Non-umami	0.034	0.897
753	AILGF	16.650	519.306	520.315	11.760		Non-umami	0.034	0.016
754	VISGI	16.460	487.301	488.310	8.730		Non-umami	0.033	0.179
755	SLGGI	20.400	445.254	446.262	7.030		Non-umami	0.033	0.028
756	LQPP		453.259	454.268	6.470	93	Non-umami	0.033	0.061
757	LPGIP	15.500	495.306	496.315	10.170		Non-umami	0.033	0.01
758	LLIAG	16.550	485.321	486.330	9.270		Non-umami	0.033	0.02
759	LGIGL	18.240	471.306	472.315	11.800		Non-umami	0.033	0.014
760	IVIIP	15.050	553.384	554.394	12.630		Non-umami	0.033	0.013
761	IVGGL	19.340	457.290	458.299	8.920		Non-umami	0.033	0.021
762	IPAGI	15.730	469.290	470.299	9.170		Non-umami	0.033	0.036
763	IGGGL	20.350	415.243	416.251	6.950		Non-umami	0.033	0.02
764	GSIII	18.680	501.316	502.325	11.050		Non-umami	0.033	0.08
765	GLALI	15.250	485.321	486.330	10.210		Non-umami	0.033	0.025
766	FPQGAP		615.302	616.312	6.510	98	Non-umami	0.033	0.017
767	ALIGF	16.650	519.306	520.315	11.760		Non-umami	0.033	0.016
768	VISGL	18.730	487.301	488.310	8.730		Non-umami	0.032	0.114
769	QPFRP		643.344	322.681	5.790	90	Non-umami	0.032	0.02
770	LILPP	16.900	551.368	552.378	10.500		Non-umami	0.032	0.011
771	IVLIP	15.050	553.384	554.394	12.630		Non-umami	0.032	0.011
772	IGGGI	20.350	415.243	416.251	6.950		Non-umami	0.032	0.028
773	GPPYLA		616.322	617.332	8.460	96	Non-umami	0.032	0.005
774	GIALL	15.250	485.321	486.330	10.210		Non-umami	0.032	0.024
775	FPFQEH		803.360	402.689	8.070	98	Non-umami	0.032	0.173
776	ASAVVGI	15.180	615.359	616.369	9.290		Non-umami	0.032	0.985
777	VPIIQ	15.110	568.358	569.368	9.390		Non-umami	0.031	0.114
778	LAIGF	17.420	519.306	520.315	11.760		Non-umami	0.031	0.014
779	KLPPP		550.348	551.356	5.240	94	Non-umami	0.031	0.012
780	IVLLP	16.080	553.384	554.394	12.630		Non-umami	0.031	0.01
781	IPAGIP	15.410	566.343	567.352	9.750		Non-umami	0.031	0.044
782	GVAPGPIW	16.720	795.428	796.438	11.350		Non-umami	0.031	0.014
783	GLIAL	15.360	485.321	486.331	10.400		Non-umami	0.031	0.025
784	GLAIL	15.250	485.321	486.330	10.210		Non-umami	0.031	0.025
785	AIIGF	16.650	519.306	520.315	11.760		Non-umami	0.031	0.018
786	LSGII	17.220	501.316	502.324	10.950		Non-umami	0.03	0.042
787	LGGGV		401.227	402.236	5.420	96	Non-umami	0.03	0.085
788	ISGIL	17.220	501.316	502.324	10.950		Non-umami	0.03	0.054
789	FPQPQP	15.450	712.354	713.365	7.740	96	Non-umami	0.03	0.009
790	AVATPVF	24.140	703.390	704.401	10.320		Non-umami	0.03	0.993
791	AIGIP	16.890	469.290	470.299	9.730		Non-umami	0.03	0.012
792	PQQIPP	19.780	678.370	679.380	6.480		Non-umami	0.029	0.139
793	LILAG	16.550	485.321	486.330	9.270		Non-umami	0.029	0.02
794	LGIGI	18.240	471.306	472.315	11.800		Non-umami	0.029	0.012
795	LGGV		344.206	345.215	5.910	97	Non-umami	0.029	0.071
796	LGGGI	20.350	415.243	416.251	6.950		Non-umami	0.029	0.026
797	LAIAI	16.900	499.337	500.346	11.530		Non-umami	0.029	0.035
798	ISGII	17.220	501.316	502.324	10.950		Non-umami	0.029	0.056
799	GPIWTP	17.040	669.349	670.359	10.420		Non-umami	0.029	0.125
800	FVVPPGHP	21.390	848.455	425.237	8.070		Non-umami	0.029	0.019
801	FPQHP		624.302	625.310	5.430	95	Non-umami	0.029	0.016
802	VLGGF	15.900	491.274	492.283	9.280		Non-umami	0.028	0.017
803	VGAGGVT	15.160	559.297	560.307	5.190		Non-umami	0.028	0.984
804	LPYP		488.264	489.273	8.520	98	Non-umami	0.028	0.012
805	LPLLP	16.050	551.368	552.378	11.970		Non-umami	0.028	0.008
806	LPLIP	16.050	551.368	552.378	11.970		Non-umami	0.028	0.009
807	LPIIP	16.050	551.368	552.378	11.970		Non-umami	0.028	0.011
808	LLEP		470.274	471.282	7.450	98	Non-umami	0.028	0.014
809	IPILP	16.050	551.368	552.378	11.970		Non-umami	0.028	0.011
810	ILVPV	15.110	539.368	540.377	12.800		Non-umami	0.028	0.009
811	IIGAN	15.430	486.280	487.288	6.650		Non-umami	0.028	0.952
812	GILAL	15.360	485.321	486.331	10.400		Non-umami	0.028	0.024
813	AGGLL	16.550	429.259	430.267	8.260		Non-umami	0.028	0.056
814	VIISGI	16.230	600.385	601.394	11.330		Non-umami	0.027	0.726
815	VIGGF	15.900	491.274	492.283	9.280		Non-umami	0.027	0.018
816	TLVVAP		598.369	599.379	8.800	97	Non-umami	0.027	0.086
817	TGGLY	15.680	509.249	510.258	5.060		Non-umami	0.027	0.773
818	PIVNP	15.520	538.312	539.321	7.750		Non-umami	0.027	0.396
819	LPGLP	15.500	495.306	496.315	10.170	98	Non-umami	0.027	0.014
820	LGAPP		453.259	454.267	6.580	96	Non-umami	0.027	0.014
821	ISGGP	15.300	429.222	430.230	4.520		Non-umami	0.027	0.038
822	FPPHT		597.291	598.299	7.230	94	Non-umami	0.027	0.186
823	PWLPP	16.860	608.332	609.342	11.640		Non-umami	0.026	0.019
824	LDVK		473.285	474.293	4.110	96	Non-umami	0.026	0.985
825	IGAIP	15.340	469.290	470.299	8.870		Non-umami	0.026	0.048
826	FGTGP		477.222	478.230	6.040	95	Non-umami	0.026	0.614
827	SIGGL	18.520	445.254	446.262	7.030		Non-umami	0.025	0.026
828	RGLP		441.270	442.278	3.570	93	Non-umami	0.025	0.03
829	LYPQQP	16.260	744.381	745.391	7.020		Non-umami	0.025	0.953
830	LGAGI	16.620	429.259	430.267	8.560		Non-umami	0.025	0.052
831	HPSLL		565.322	566.332	7.040	91	Non-umami	0.025	0.353
832	AAAVP	15.780	427.243	428.252	5.750		Non-umami	0.025	0.466
833	TIGGL	19.510	459.269	460.278	8.680		Non-umami	0.024	0.092
834	TFPHQP	20.390	725.350	363.683	5.840		Non-umami	0.024	0.188
835	QGGLL	15.000	486.280	487.289	8.300		Non-umami	0.024	0.262
836	PGAYPGAP	18.280	728.349	729.359	6.250		Non-umami	0.024	0.054
837	LPLP		438.284	439.293	10.000	98	Non-umami	0.024	0.01
838	IAAGP	15.080	427.243	428.251	5.260		Non-umami	0.024	0.045
839	FLPQLP	16.410	713.411	714.421	11.930	96	Non-umami	0.024	0.01
840	AASL		360.201	361.209	4.620	92	Non-umami	0.024	0.835
841	VPGGL	17.030	441.259	442.267	7.360	99	Non-umami	0.023	0.277
842	VLGGL	17.100	457.290	458.299	8.920		Non-umami	0.023	0.056
843	PFLQPHQP	16.830	962.497	482.258	7.930		Non-umami	0.023	0.028
844	LPYPQP	21.350	713.375	714.385	8.580	91	Non-umami	0.023	0.02
845	LIPTH	15.720	579.338	580.346	7.200		Non-umami	0.023	0.057
846	LGGAP		413.227	414.236	4.160	92	Non-umami	0.023	0.129
847	IVILP	15.050	553.384	554.394	12.630		Non-umami	0.023	0.012
848	IAIAI	16.900	499.337	500.346	11.530		Non-umami	0.023	0.04
849	GPAYP	15.520	503.238	504.247	5.700		Non-umami	0.023	0.039
850	GLIAI	15.360	485.321	486.331	10.400		Non-umami	0.023	0.027
851	GIIAI	15.360	485.321	486.331	10.400		Non-umami	0.023	0.044
852	GIAII	15.250	485.321	486.330	10.210		Non-umami	0.023	0.044
853	FLPQIP	16.410	713.411	714.421	11.930		Non-umami	0.023	0.013
854	YPAGP		503.238	504.247	5.850	98	Non-umami	0.022	0.056
855	YGGAP	15.710	463.207	464.215	3.390	98	Non-umami	0.022	0.072
856	VIGGL	16.850	457.290	458.299	8.920		Non-umami	0.022	0.021
857	PRPP		465.270	466.279	6.950	96	Non-umami	0.022	0.014
858	LVLIP	16.080	553.384	554.394	12.630		Non-umami	0.022	0.01
859	LVIIP	16.080	553.384	554.394	12.630		Non-umami	0.022	0.012
860	FVHP		498.259	499.267	5.530	97	Non-umami	0.022	0.019
861	VPGGI	16.970	441.259	442.267	7.360		Non-umami	0.021	0.012
862	PGAYP	15.200	503.238	504.247	5.700		Non-umami	0.021	0.041
863	LPQPP		550.312	551.321	7.710	97	Non-umami	0.021	0.036
864	LPILP	16.050	551.368	552.378	11.970		Non-umami	0.021	0.01
865	LIIAG	16.550	485.321	486.330	9.270		Non-umami	0.021	0.035
866	ILIAG	16.550	485.321	486.330	9.270		Non-umami	0.021	0.023
867	GILAI	15.360	485.321	486.331	10.400		Non-umami	0.021	0.027
868	FPQQP	15.710	615.302	616.310	6.600	95	Non-umami	0.021	0.972
869	ERVW		588.302	589.312	6.150	97	Non-umami	0.021	0.04
870	AVLGI	17.090	471.306	472.315	11.800		Non-umami	0.021	0.011
871	AVIGL	17.090	471.306	472.315	11.800		Non-umami	0.021	0.011
872	TGGHFP	16.640	614.281	615.291	5.920		Non-umami	0.02	0.149
873	SIGGI	20.400	445.254	446.262	7.030		Non-umami	0.02	0.037
874	QPQFPP	16.550	712.354	713.363	7.230		Non-umami	0.02	0.053
875	QGGLI	15.000	486.280	487.289	8.300		Non-umami	0.02	0.108
876	LPQFPHPQ	16.110	962.497	482.258	7.930		Non-umami	0.02	0.055
877	VIGGI	17.100	457.290	458.299	8.920		Non-umami	0.019	0.027
878	PQFPQPP	15.480	809.407	810.419	8.940		Non-umami	0.019	0.02
879	PFLQPHQ	19.130	865.445	433.731	7.600		Non-umami	0.019	0.993
880	LVILP	16.080	553.384	554.394	12.630		Non-umami	0.019	0.011
881	GIIAL	15.360	485.321	486.331	10.400		Non-umami	0.019	0.041
882	GIALI	15.250	485.321	486.330	10.210		Non-umami	0.019	0.027
883	GIAIL	15.250	485.321	486.330	10.210		Non-umami	0.019	0.038
884	FPQQPP	18.860	712.354	713.363	7.150		Non-umami	0.019	0.273
885	FPPQQP	20.980	712.354	713.363	6.970		Non-umami	0.019	0.261
886	EIGGI	15.930	487.264	488.273	7.060		Non-umami	0.019	0.861
887	AVIGI	17.090	471.306	472.315	11.800		Non-umami	0.019	0.012
888	VLGGI	17.100	457.290	458.299	8.920		Non-umami	0.018	0.02
889	TIGGI	17.870	459.269	460.278	8.680		Non-umami	0.018	0.047
890	QGGII	15.000	486.280	487.289	8.300		Non-umami	0.018	0.11
891	LIVPV	15.090	539.368	540.377	12.800		Non-umami	0.018	0.01
892	LGVL		400.269	401.277	9.600	93	Non-umami	0.018	0.008
893	IIPQQP	17.010	694.401	695.410	7.170		Non-umami	0.018	0.045
894	YYQPPR		822.402	412.210	5.220	97	Non-umami	0.017	0.945
895	VVLF		476.300	477.309	11.610	91	Non-umami	0.017	0.015
896	QLGL		429.259	430.267	9.170	94	Non-umami	0.017	0.352
897	ALGW		445.233	446.241	9.470	90	Non-umami	0.017	0.016
898	AGGIL	16.550	429.259	430.267	8.260		Non-umami	0.017	0.021
899	YPQQPIP	17.460	841.433	842.443	9.410		Non-umami	0.016	0.027
900	TGGIY	16.400	509.249	510.258	5.060		Non-umami	0.016	0.307
901	QGGIL	15.000	486.280	487.289	8.300		Non-umami	0.016	0.103
902	VVPPGHP	24.980	701.386	351.701	5.410	92	Non-umami	0.015	0.01
903	KLGL		471.306	472.315	11.800	95	Non-umami	0.015	0.018
904	IGAGL	16.620	429.259	430.267	8.560		Non-umami	0.015	0.043
905	FPTH		500.238	501.247	6.760	92	Non-umami	0.015	0.279
906	IGAGI	16.620	429.259	430.267	8.560		Non-umami	0.014	0.058

**Table A2:** Multi-dimensional sensory evaluation of lager beer.

Different Samples	Aroma Intensity ^b^	Malt Aroma ^c^	Hop Aroma ^a^	Fermentation-Derived (By-Product) Aroma ^a^	Sweet Taste ^e^	Bitterness ^i^	Umami Taste ^h^	Carbonic Bite ^f^	Smoothness ^f^	Bitterness Persistence ^c^	Malt/Hop Aftertaste ^b^	Residual Off-Flavour ^g^	Overall Balance and Typicity ^d^
A_a_	7.05 + 1.79	7.45 + 1.93	7.00 + 2.13	7.05 + 1.70	6.95 + 1.9	6.50 + 2.06	6.30 + 2.03	7.00 + 1.86	7.30 + 1.75	7.50 + 2.35	7.40 + 1.98	7.05 + 2.33	7.50 + 1.50
A-1_c_	7.30 + 1.66	7.75 + 1.62	7.25 + 1.74	6.95 + 1.32	8.05 + 1.9	6.90 + 1.48	7.65 + 1.90	8.00 + 1.72	7.70 + 1.84	7.80 + 1.70	7.00 + 1.52	8.25 + 1.62	7.85 + 1.84
A-2_b_	7.55 + 1.76	7.15 + 1.63	7.20 + 1.77	7.50 + 1.82	7.05 + 1.85	7.30 + 1.81	7.70 + 1.78	7.40 + 2.06	7.90 + 1.74	7.10 + 1.55	7.45 + 1.64	8.35 + 1.69	7.55 + 1.32
A-3_d_	6.25 + 0.79	5.90 + 0.85	5.65 + 0.67	5.85 + 0.81	5.95 + 0.94	5.80 + 0.89	6.45 + 1.67	6.00 + 0.79	6.05 + 0.83	6.40 + 0.82	6.00 + 0.86	5.90 + 0.91	6.15 + 0.88
A-4_d_	6.00 + 0.65	5.60 + 0.75	6.20 + 0.77	5.90 + 0.79	6.10 + 0.97	5.80 + 0.89	6.40 + 1.27	6.40 + 0.68	6.30 + 0.8	6.00 + 0.86	6.00 + 0.86	6.25 + 0.79	5.80 + 0.77
A-5_b_	7.75 + 1.52	7.05 + 1.67	7.40 + 1.85	7.30 + 2.00	7.55 + 1.70	7.30 + 1.87	7.35 + 1.60	7.95 + 1.43	7.45 + 1.64	7.10 + 1.65	7.30 + 1.56	7.70 + 1.81	7.50 + 1.70
A-6_a_	7.20 + 1.77	7.05 + 2.39	6.80 + 2.31	7.10 + 1.92	6.90 + 1.71	5.90 + 1.74	7.00 + 2.71	6.25 + 2.29	6.20 + 1.74	7.15 + 2.06	6.50 + 1.61	6.65 + 1.98	7.25 + 1.89

Note: Symbols such as a, b, and c represent the significance between different samples and sensory dimensions.
